# Oncogenic *SLC2A11–MIF* fusion protein interacts with polypyrimidine tract binding protein 1 to facilitate bladder cancer proliferation and metastasis by regulating mRNA stability

**DOI:** 10.1002/mco2.685

**Published:** 2024-08-14

**Authors:** Liang Cheng, Chenwei Yang, Junlin Lu, Ming Huang, Ruihui Xie, Sarah Lynch, Justin Elfman, Yuhang Huang, Sen Liu, Siting Chen, Baoqing He, Tianxin Lin, Hui Li, Xu Chen, Jian Huang

**Affiliations:** ^1^ Department of Urology Sun Yat‐sen Memorial Hospital, Sun Yat‐sen University Guangzhou Guangdong China; ^2^ Guangdong Provincial Key Laboratory of Malignant Tumor Epigenetics and Gene Regulation Department of Urology,Sun Yat‐sen Memorial Hospital,Sun Yat‐Sen University Guangzhou Guangdong China; ^3^ Guangdong Provincial Clinical Research Center for Urological Diseases Department of Urology, Sun Yat‐sen Memorial Hospital, Sun Yat‐sen University Guangzhou Guangdong China; ^4^ Department of Pathology School of Medicine University of Virginia Charlottesville Virginia USA

**Keywords:** bladder cancer, fusion protein, metastasis, mRNA stability, *PTBP1*, *SLC2A11*–*MIF*

## Abstract

Chimeric RNAs, distinct from DNA gene fusions, have emerged as promising therapeutic targets with diverse functions in cancer treatment. However, the functional significance and therapeutic potential of most chimeric RNAs remain unclear. Here we identify a novel fusion transcript of solute carrier family 2‐member 11 (*SLC2A11*) and macrophage migration inhibitory factor (*MIF*). In this study, we investigated the upregulation of *SLC2A11–MIF* in The Cancer Genome Atlas cohort and a cohort of patients from Sun Yat‐Sen Memorial Hospital. Subsequently, functional investigations demonstrated that *SLC2A11–MIF* enhanced the proliferation, antiapoptotic effects, and metastasis of bladder cancer cells in vitro and in vivo. Mechanistically, the fusion protein encoded by *SLC2A11–MIF* interacted with polypyrimidine tract binding protein 1 (*PTBP1*) and regulated the mRNA half‐lives of Polo Like Kinase 1, Roundabout guidance receptor 1, and phosphoinositide‐3‐kinase regulatory subunit 3 in BCa cells. Moreover, *PTBP1* knockdown abolished the enhanced impact of *SLC2A11–MIF* on biological function and mRNA stability. Furthermore, the expression of *SLC2A11–MIF* mRNA is regulated by CCCTC‐binding factor and stabilized through RNA N4‐acetylcytidine modification facilitated by N‐acetyltransferase 10. Overall, our findings revealed a significant fusion protein orchestrated by the *SLC2A11–MIF–PTBP1* axis that governs mRNA stability during the multistep progression of bladder cancer.

## Graphical abstract

Chimeric RNA *SLC2A11–MIF* is upregulated and encodes a fusion protein in bladder cancer. The fusion protein *SLC2A11–MIF* localizes to the cytoplasm and interacts with *PTBP1* to regulate the mRNA stability of *PLK1*, *ROBO1*, and *PIK3R3*. This ultimately leads to upregulation of these oncogenes, thereby controlling proliferation and metastasis. Additionally, *SLC2A11–MIF* mRNA is further regulated by *CTCF* and stabilized through ac4C modification facilitated by *NAT10*. Targeting *SLC2A11–MIF* represents a promising and versatile therapeutic strategy for patients with bladder cancer.

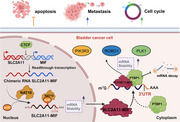

## 1 INTRODUCTION

Chimeric RNAs are composite transcripts comprising exons from distinct genes at the RNA level.^1^, ^2^ These alterations are conventionally attributed to fusion genes resulting from chromosomal rearrangements.^3^, ^4^ However, advancements in deep sequencing technologies^5^, ^6^ have resulted in the identification of noncanonical chimeric RNAs that arise from intergenic splicing events without any alterations at the DNA level.^7^ Trans‐splicing or cis‐splicing events between adjacent genes (cis‐SAGe) represents a pivotal mechanism underlying the generation of chimeric transcripts.^8^, ^9^ Further investigation revealed that chimeric RNAs play a significant role independent of their parental genes.^10^, ^11^ However, the complete elucidation of the biological role and molecular mechanism underlying chimeric RNAs remains to be achieved.

Since the discovery of *GOLM1–NAA35* in esophageal carcinoma, there has been a substantial demand for potential diagnostic and therapeutic targets pertaining to cancer‐specific chimeric RNAs.^12^ Subsequently, additional noncanonical chimeric RNAs, such as *SLC45A3–ELK4* in prostate cancer,^13^, ^14^ *RRM2–c2orf48* in nasopharyngeal carcinoma and lung cancer,^15^, ^16^ and *BCL2L2–PABPN1* in glioblastoma and bladder cancer (BCa),^2^, ^11^ have been reported in various solid cancers. These chimeric RNAs have demonstrated their importance in tumor growth, metastasis, drug resistance, and signaling pathway activation.^17^ However, unravelling the expression patterns and biological functions underlying chimeric RNAs in BCa remains a persistent challenge that requires further investigation.

Chimeric RNAs can exert a diverse range of cellular effects through multiple mechanisms, including acting as long noncoding RNAs,^13^ fusion proteins,^18^ and perturbing the expression of parental genes.^19^ In prostate cancer, *SLC45A3–ELK4* serves as a long noncoding RNA to regulate tumor proliferation.^13^ *RAD51AP1–DYRK4*, on the other hand, encodes a fusion protein that triggers the activation of the MEK/ERK signaling pathway to enhance sensitivity to trametinib.^20^ A recently discovered chimeric RNA called *SLC2A11–MIF* has exhibited a high prevalence in cervical,^21^ colorectal,^22^ and bladder cancer.^2^ However, the precise molecular mechanisms underlying the activity of *SLC2A11–MIF* remain elusive.

In our study, we focused on the highly prevalent chimeric RNA *SLC2A11–MIF* that facilitates the proliferation and metastasis of bladder cancer cells in vitro and enhances tumor growth and lymphatic metastasis in vivo. Mechanistically, *SLC2A11–MIF* recruited *PTBP1* to stabilize Polo Like Kinase 1 (*PLK1*), Roundabout guidance receptor 1 (*ROBO1*), and phosphoinositide‐3‐kinase regulatory subunit 3 (*PIK3R3*) mRNA, thereby upregulating their expression levels. Additionally, we discovered that *SLC2A11–MIF* is generated as a byproduct of readthrough transcription and is regulated by CCCTC‐binding factor (*CTCF*) and N‐acetyltransferase 10 (*NAT10*). Consequently, targeting *SLC2A11–MIF* could serve as a therapeutic strategy for reducing the proliferation and metastasis of bladder cancer.

## 2 RESULTS

### 2.1 Characterization of the chimeric RNA *SLC2A11–MIF* and its parental genes in bladder cancer

**FIGURE 1:**
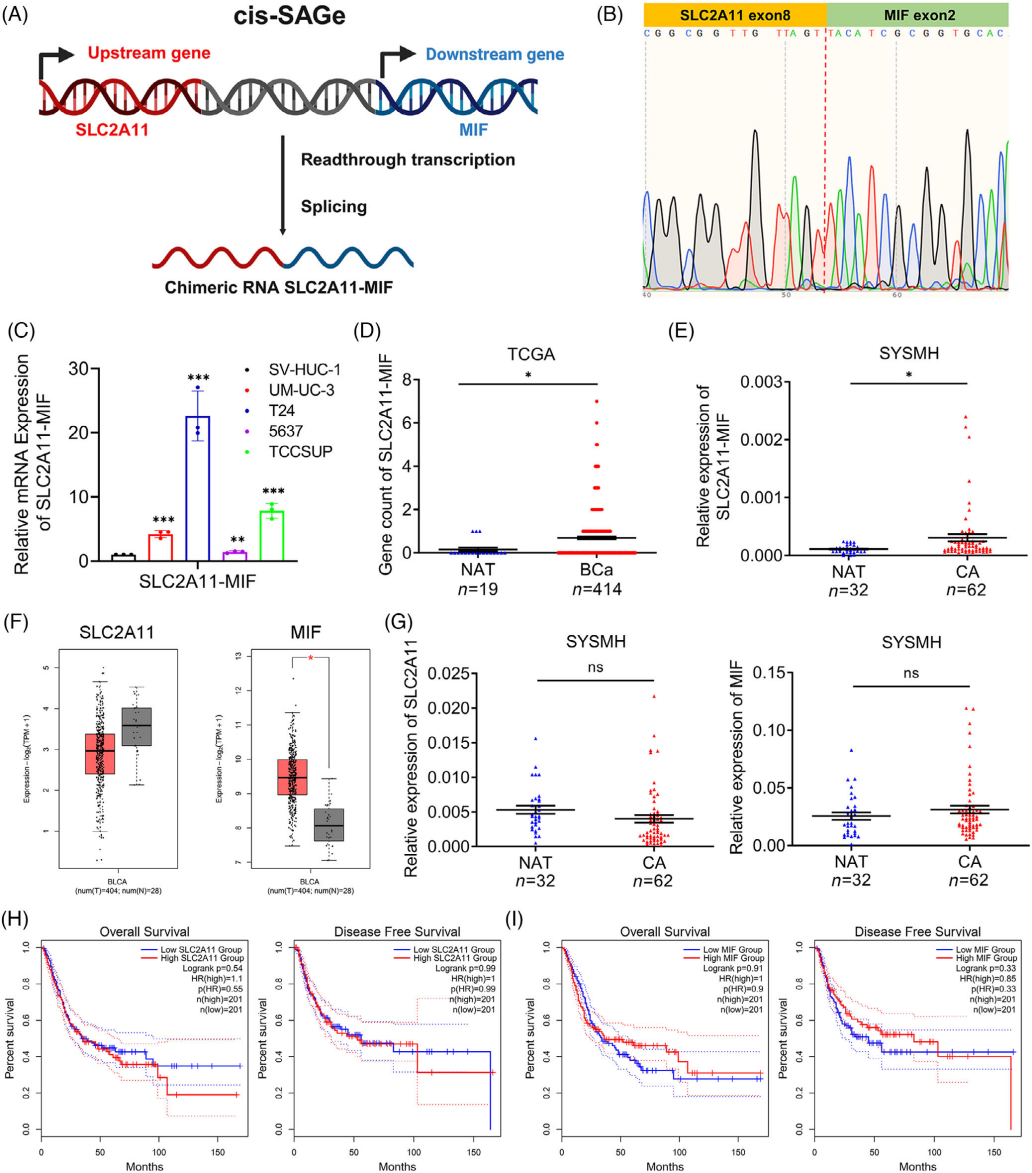
Characterization of the chimeric RNA *SLC2A11–MIF* and its parental genes in bladder cancer. (A) Mechanisms of chimeric RNA *SLC2A11–MIF* formation. Red or blue segments represent exons, and gray segments represent introns or intergenic regions. Cis‐splicing occurs between adjacent genes. Transcriptional readthrough occurs between *SLC2A11* and *MIF* on the same strand with identical transcriptional orientation. (B) Sanger sequencing results of *SLC2A11–MIF*, with the junction indicated by a red dashed line. (C) *SLC2A11–MIF* mRNA expression in human uroepithelial cells, SV‐HUC‐1 cells, and 4 bladder cancer cell lines, UM‐UC‐3, T24, 5637, and TCCSUP cells. (D) The gene counts of *SLC2A11–MIF* in NATs and BCa tissues were analyzed using data from the TCGA database. (E) The expression of *SLC2A11–MIF* was detected in bladder cancer tissues paired with NATs in the SYSMH cohort. (F) The expression of parental genes in BCa tissues paired with normal adjacent normal tissues in the TCGA cohort was detected. (G) The expression of parental genes was measured by qRT‐PCR in 94 BCa tissues paired with NATs. (H and I) Kaplan–Meier curves for OS and DFS of patients with bladder cancer with high versus low expression of parental genes. *p < 0.05, **p < 0.01, ***p < 0.001, ns indicates not statistically significant.

Chimeric RNA *SLC2A11–MIF* is classified as a read‐through event occurring between adjacent genes transcribed on the same DNA strand, was confirmed through Sanger sequencing (Figure 1A,B). Our findings demonstrated the significant upregulation of *SLC2A11–MIF* in BCa cell lines (Figure 1C), and this observation was further supported by The Cancer Genome Atlas (TCGA) data and an independent 94‐case cohort from Sun Yat‐Sen Memorial Hospital (SYSMH) (Figure 1D,E). We also investigated the expression of the parental genes *SLC2A11* and *MIF* in the TCGA and SYSMH cohorts (Figure 1F,G). Furthermore, there was no significant difference in overall survival (OS) or disease‐free survival (DFS) among patients displaying elevated levels of *SLC2A11* and *MIF* expression (Figure 1H,I). In conclusion, these results suggest that the upregulation of the *SLC2A11–MIF* transcript is a prevalent characteristic of bladder cancer.

### 2.2 Knockdown of *SLC2A11–MIF* inhibited proliferation in vitro and tumor growth in vivo

**FIGURE 2:**
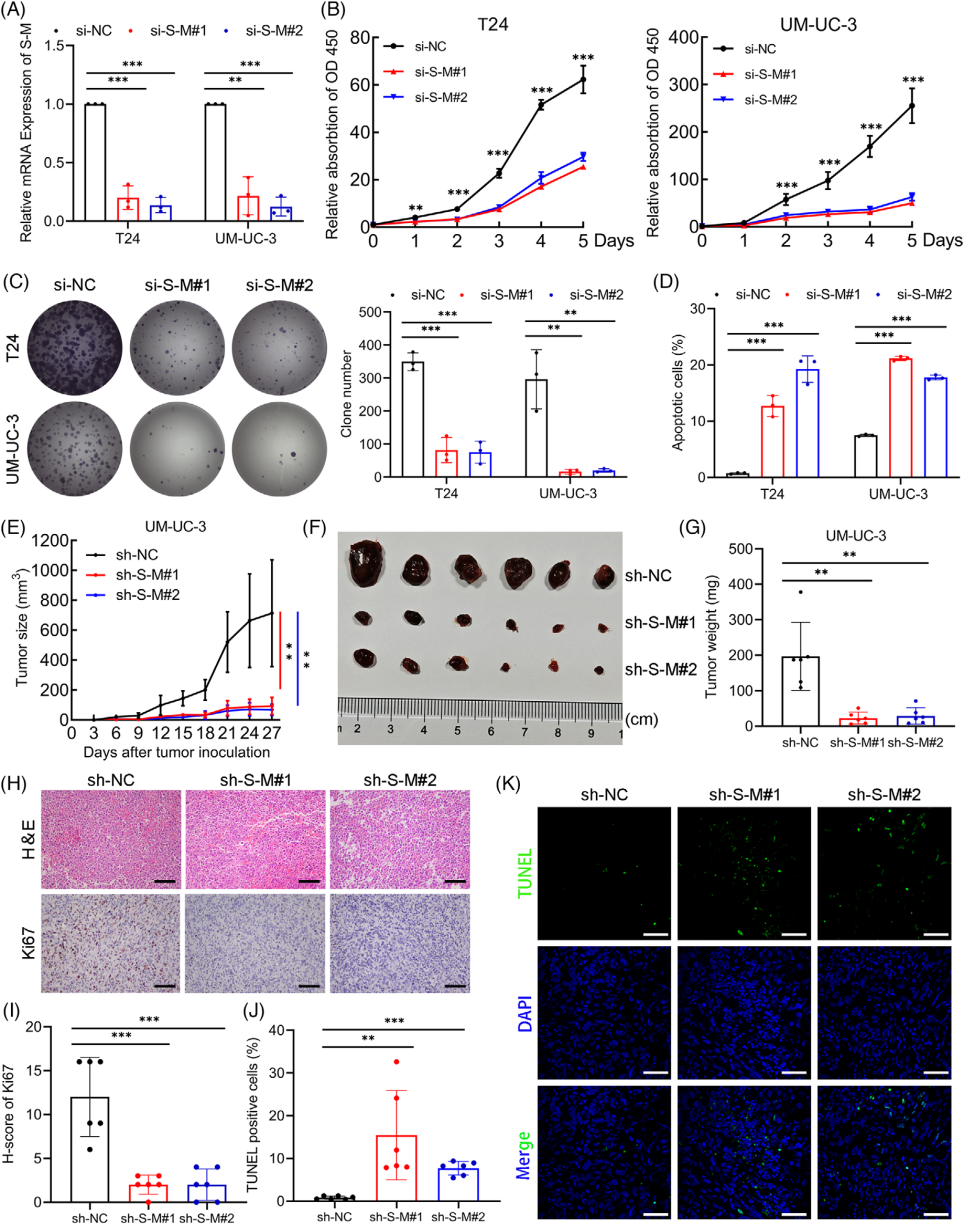
Knockdown of *SLC2A11–MIF* inhibited proliferation in vitro and tumor growth in vivo. (A) qRT‐PCR analysis was conducted to evaluate the expression of *SLC2A11–MIF* in *SLC2A11–MIF*‐silenced and control BCa cells. (B) Cell viability determined by a CCK‐8 assay was evaluated in T24 and UM‐UC‐3 cells with *SLC2A11–MIF* knockdown. (C) Colony formation assays were performed in T24 and UM‐UC‐3 cells with *SLC2A11–MIF* knockdown. (D) Quantification of cell apoptosis in T24 and UM‐UC‐3 cells with knockdown of *SLC2A11–MIF*. (E) The growth of UM‐UC‐3 tumors with *SLC2A11–MIF* knockdown was assessed every 3 days, and tumor growth curves were generated. The mean and standard deviation (SD) of the tumor volumes measured in six mice are shown. (F) Representative image of subcutaneous tumors in which *SLC2A11–MIF* knockdown is presented. (G) Weight (mg) of tumors with *SLC2A11–MIF* knockdown after surgical dissection. (H) Representative images of H&E and IHC staining demonstrating Ki67 expression in tumors. Scale bars: 100 µm (black). (I) Histogram of the *H*‐scores of Ki67 cells with *SLC2A11–MIF* knockdown. (J and K) Representative images and histograms showing the proportions of TUNEL‐positive cells. Scale bars: 50 µm (white). **p < 0.01, ***p < 0.001.

To explore the impact of *SLC2A11–MIF* on bladder cancer progression, we utilized specific small interfering RNAs (siRNAs) to effectively suppress *SLC2A11–MIF* expression. Quantitative reverse transcription PCR (qRT‐PCR) analysis revealed significant downregulation of *SLC2A11–MIF*, while the expression of the parental genes *SLC2A11* and *MIF* was unaltered (Figures 2A and S1A). Subsequently, using a Cell Counting Kit‐8 (CCK‐8) and colony formation assays, we demonstrated that *SLC2A11–MIF* knockdown significantly decreased cell viability (Figure 2B,C). Additionally, flow cytometric analysis revealed that knockdown of *SLC2A11–MIF* resulted in a significant increase in apoptosis, accompanied by cell cycle arrest at the G0/G1 phase and a corresponding decrease in the population within the S phase (Figures 2D and S1B–D). Subsequently, we established stable *SLC2A11–MIF*‐silenced or nontargeting control UM‐UC‐3 cells and administered them via subcutaneous injection into BALB/c nude mice (Figure S2A). Furthermore, knockdown of *SLC2A11–MIF* led to a significant decrease in tumor growth (Figure 2E) and reduced tumor size and weight (Figure 2F,G). Moreover, the tumors derived from cells with suppressed *SLC2A11–MIF* exhibited reduced levels of Ki67, a marker associated with cellular proliferation (Figure 2H,I), and an increased proportion of TUNEL‐positive cells (Figure 2J,K). Collectively, these findings strongly indicate that *SLC2A11–MIF* promotes cell proliferation by modulating both apoptotic pathways and the G1/S phase transition in vitro and in vivo.

### 2.3 Knockdown of *SLC2A11–MIF* suppressed metastasis independent of its parental genes in vitro and lymphatic metastasis in vivo

**FIGURE 3:**
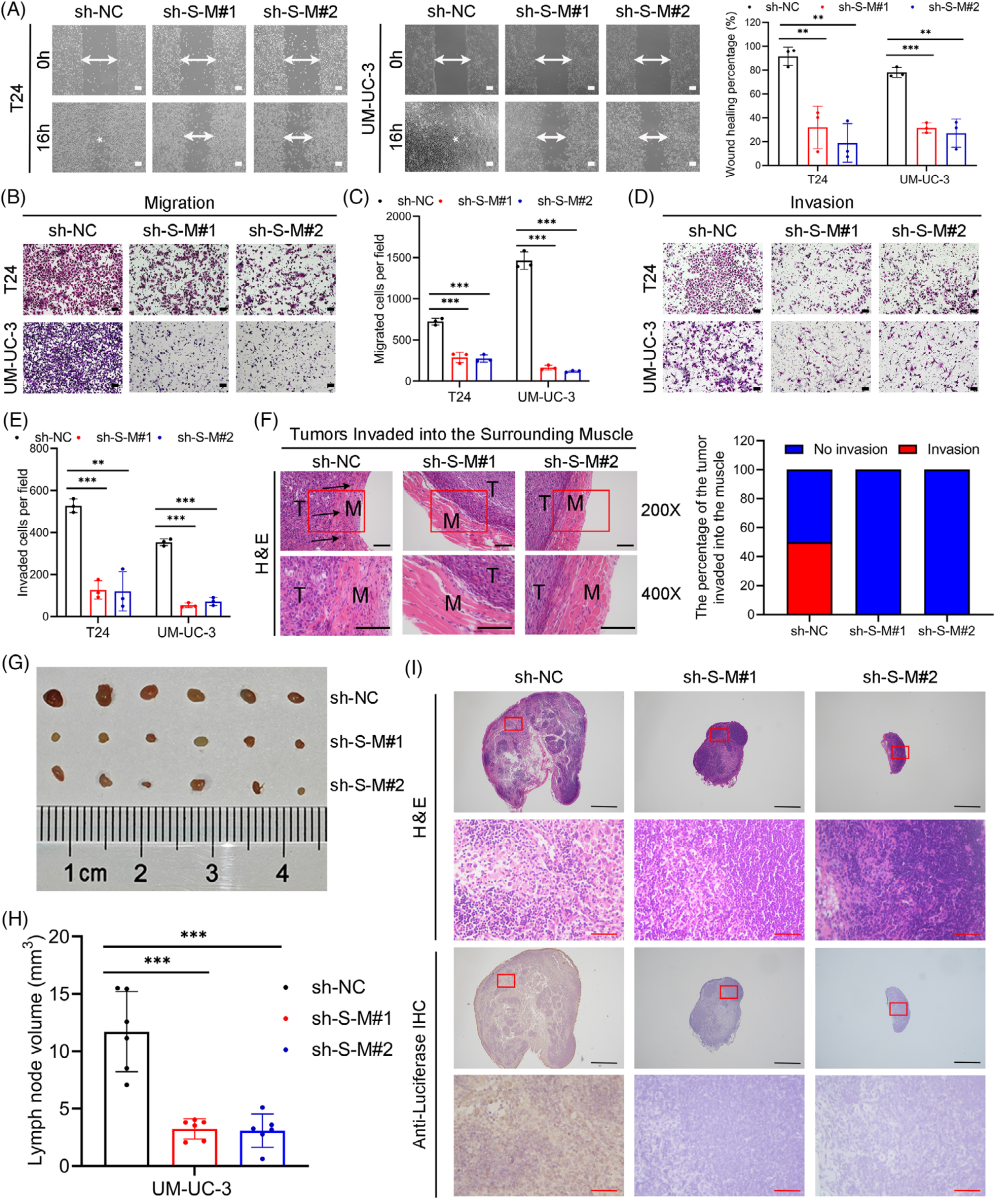
Knockdown of *SLC2A11–MIF* suppressed the metastatic behavior of bladder cancer cells in vitro and lymphatic metastasis in vivo. (A) Representative images and histograms of wound healing assays in T24 and UM‐UC‐3 cells demonstrating decreased cellular motility following knockdown of *SLC2A11–MIF*. (B and C) Representative images and histograms of migration assays in T24 and UM‐UC‐3 cells reveal decreased cell migratory capacity after knockdown of *SLC2A11–MIF*. (D and E) Representative images and histograms illustrating the invasion of T24 and UM‐UC‐3 cells after silencing *SLC2A11–MIF*. (F) Representative images showing tumor invasion into the surrounding muscle in the *SLC2A11–MIF* knockdown groups compared with the respective controls. M represents muscle, while T represents tumor. Arrows indicate invasive tissues. The histogram shows the percentage of tumor invasion into the surrounding muscle between the *SLC2A11–MIF*‐knockdown group and the control group. (G and H) Representative images of dissected popliteal LNs and histogram analysis of the LN volume. (I) Representative images of H&E and IHC staining for LN status. Scale bars, 500 mm (black) and 50 mm (red). **p < 0.01, ***p < 0.001.

To evaluate the impact of *SLC2A11–MIF* on metastatic behavior, we conducted wound healing, migration, and invasion assays. Furthermore, knockdown of *SLC2A11–MIF* effectively suppressed the migration and invasion of BCa cells (Figure 3A–E). Importantly, our findings revealed that control BCa cells formed tumors characterized by invasive spike‐like structures penetrating the surrounding muscle tissues, whereas *SLC2A11–MIF*‐knockdown cells exhibited distinct smooth edges (Figure 3F). Subsequently, we established a nude mouse model by injecting UM‐UC‐3/luciferase into the footpads in vivo (Figure S2B). Remarkably, mice bearing *SLC2A11–MIF*‐knockdown tumors exhibited prolonged survival (Figure S2C). Importantly, the incidence of lymphatic metastasis significantly decreased from 66.67% in the control group to 16.67 and 0% in the *SLC2A11–MIF* knockdown group (Figure S2D). Furthermore, the popliteal lymph nodes of the *SLC2A11–MIF* shRNA‐treated mice exhibited significantly reduced volumes (Figure 3G,H). The presence of metastatic LNs was confirmed through H&E staining as well as immunostaining for luciferase (Figure 3I). The collective findings of this investigation demonstrate that *SLC2A11–MIF* plays a pivotal role in facilitating the migration and invasion of BCa cells in vitro and lymphatic metastasis in vivo.

To investigate the distinct functions of *SLC2A11–MIF* independent of their parental genes, we used specific siRNAs to knockdown the expression of parental genes while keeping the chimeric expression levels of *SLC2A11–MIF* unaltered (Figure S3A–D). Subsequently, we demonstrated that knockdown of both *SLC2A11* and *MIF* significantly impaired the viability and colony‐forming ability (Figure S3E,F). However, knockdown of the parental genes did not significantly affect the migration or invasion of the BCa cells (Figure S3G–K). Therefore, these findings provide evidence for the distinct metastatic function of *SLC2A11–MIF* independent of its parental genes in vitro.

### 2.4 Characterization of the *SLC2A11–MIF* encoded fusion protein in BCa

**FIGURE 4:**
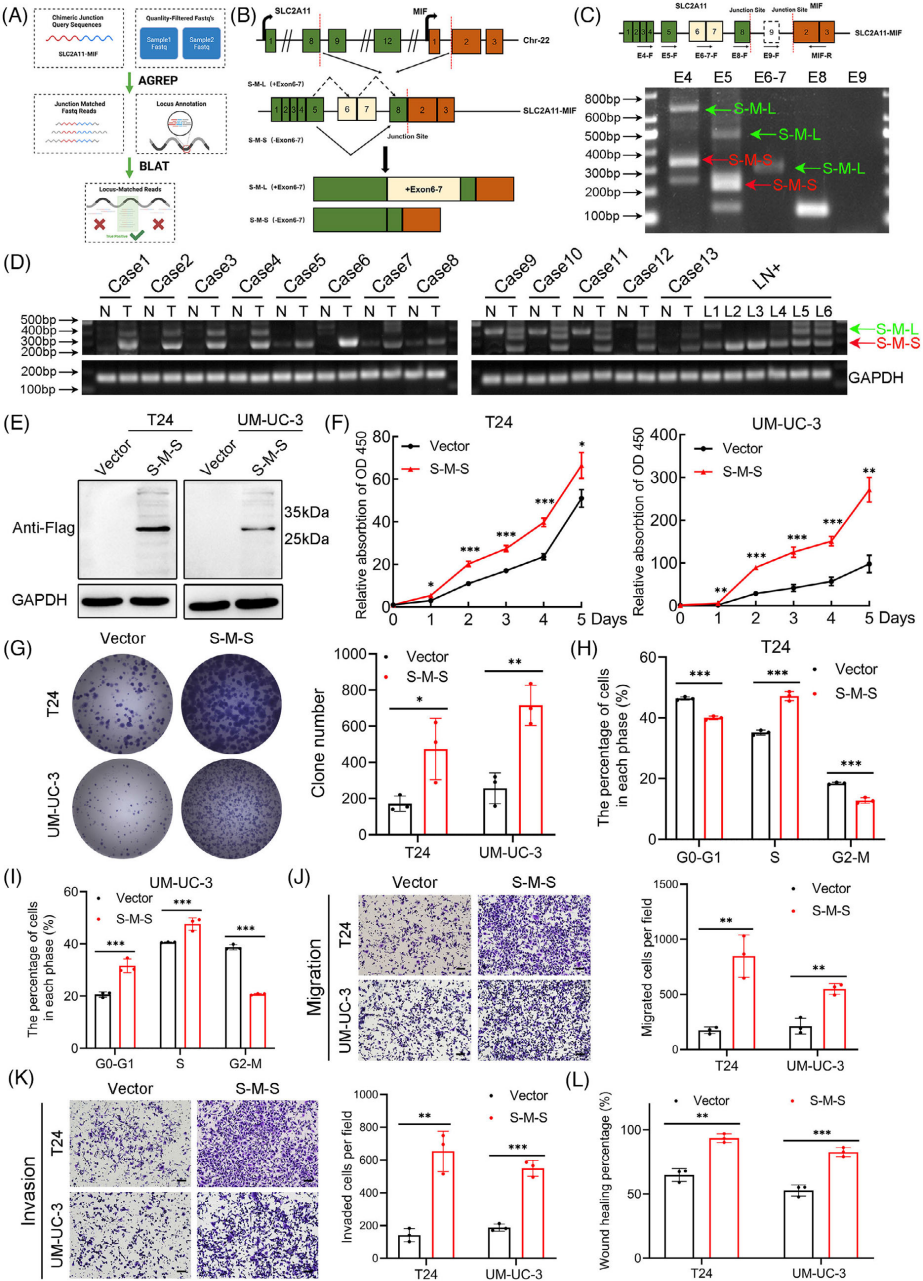
Characterization and functional analysis of the *SLC2A11–MIF* fusion protein. (A) The flowchart illustrates the comprehensive analysis of full‐length *SLC2A11–MIF*. (B) Structures of the two isoforms of the *SLC2A11–MIF* fusion are depicted, with blocks representing exons and lines representing introns or the intergenic region. (C) Top: Diagram showing the variants of *SLC2A11–MIF* mRNA and the primers used for RT‐PCR detection of exon 4 (primer E4), exons 5 (primer E5), exons 6 and 7 (primer E6‐7), exons 8 (primer E8), and exons 9 (primer E9). Bottom: Expression levels of S‐M‐L and S‐M‐S isoforms were assessed using RT‐PCR in UM‐UC‐3 cells. (D) Representative images demonstrating the presence of S‐M‐L and S‐M‐S by RT‐PCR in BCa tissues and metastatic lymph node (LN+) tissues. (E) Western blotting was performed to determine S‐M‐S‐FLAG expression levels in S‐M‐S‐overexpressing cells and control cells. (F) Cell viability of S‐M‐S‐overexpressing T24 and UM‐UC‐3 cells. (G) Colony formation in S‐M‐S‐overexpressing T24 and UM‐UC‐3 cells. (H and I) The percentages (%) of cell populations at different stages of the cell cycle are provided for *SLC2A11–MIF*‐overexpressing T24 and UM‐UC‐3 cells. (J) Representative images and histograms of migration assays in T24 and UM‐UC‐3 cells reveal increased cell migratory capacity after overexpressing S‐M‐S. Scale bars: black, 100 µm. (K) Representative images and histograms illustrating the invasion of T24 and UM‐UC‐3 cells overexpressing S‐M‐S. Scale bars: black, 100 µm. (L) Histograms from wound healing assays demonstrating cellular motility following overexpression of S‐M‐S in T24 and UM‐UC‐3 cells. Scale bars: black, 100 µm. *p < 0.05, **p < 0.01, ***p < 0.001.

Expanding upon our previous investigation, we identified two predominant isoforms of *SLC2A11–MIF* using the AGREP methodology (Figure 4A). One variant involves the fusion of exon 8 from *SLC2A11* with exon 2 from *MIF* (referred to as S‐M‐L), while the other isoform, denoted as e5‐e8‐e2 (S‐M‐S), arises from the exclusion of exons 6 and 7 followed by the joining of exon 5 with exon 8 from *SLC2A11* before merging with exon 2 of *MIF* (Figure 4B). Subsequently, we designed exon‐specific primers flanking the chimeric junction site and confirmed the expression of both isoforms of *SLC2A11–MIF* by RT‐PCR and Sanger sequencing and observed increased expression of the S‐M‐S isoform (Figures 4C and S4A‒C). Notably, S‐M‐S exhibited the most significant upregulation in both BCa tissues and metastatic lymph node tissues (LN+) compared with NATs (Figure 4D). To evaluate the translatability of *SLC2A11–MIF* transcripts, we generated predominant variant S‐M‐S open reading frame (ORF) in T24 and UM‐UC‐3. qRT‐PCR and western blot analysis revealed specific protein bands corresponding to S‐M‐S in transduced T24 and UM‐UC‐3 cells (Figures 4E and S4D). The subcellular localization of the fusion protein is strongly associated with its underlying biological mechanism, as evidenced by western blot and immunofluorescence analyses revealing a predominantly cytoplasmic distribution of the *SLC2A11–MIF* fusion protein (Figure S4E,F). In conclusion, these results demonstrated that the S‐M‐S fusion transcript is the predominant isoform and encodes a fusion protein in BCa cells.

### 2.5 *SLC2A11–MIF* enhanced the growth and metastasis of BCa in vitro and in vivo

**FIGURE 5:**
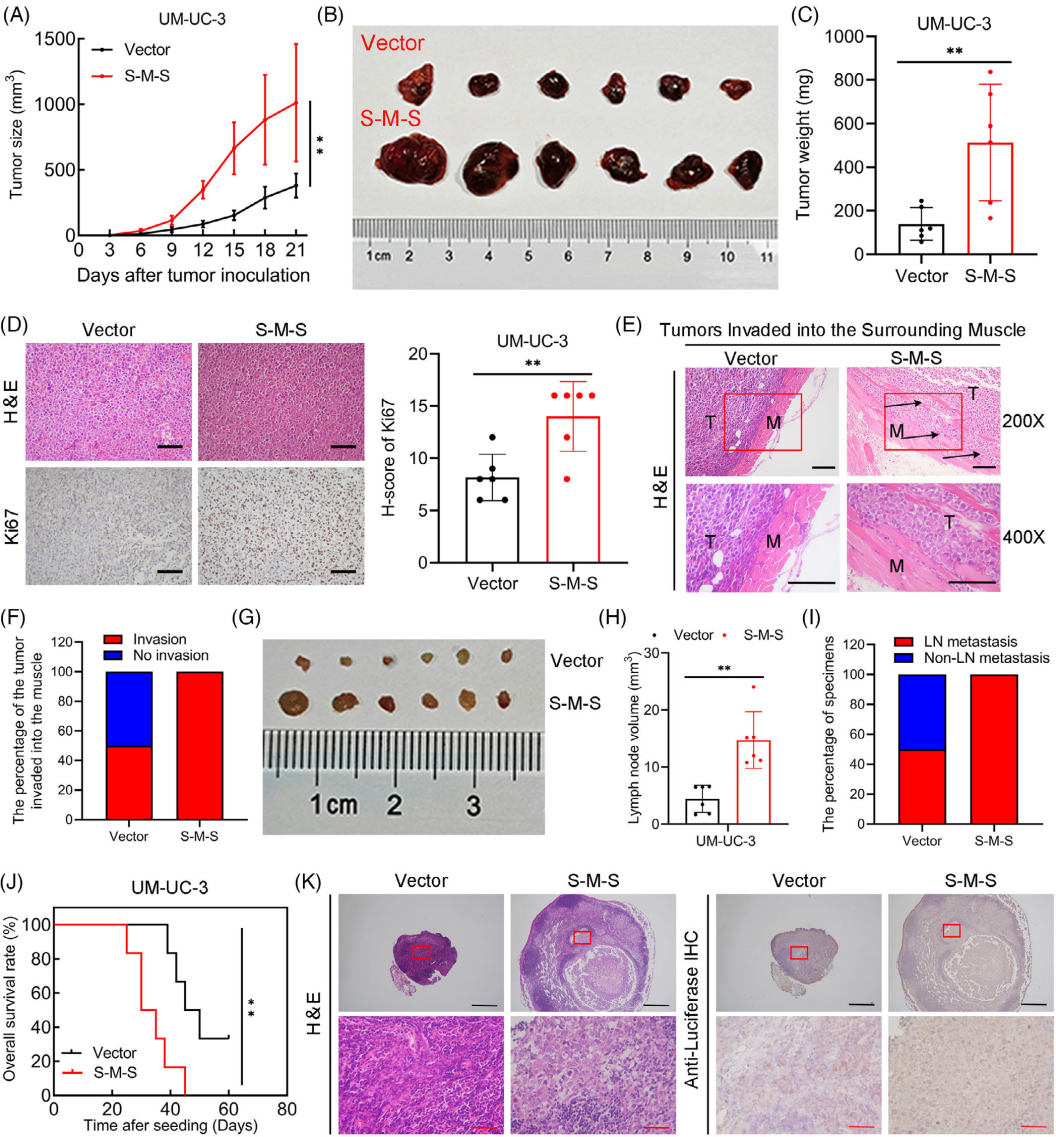
*SLC2A11–MIF* enhanced the proliferation and metastasis of BCa cells in vivo. (A) Growth curves of UM‐UC‐3 tumors with control or *SLC2A11–MIF* overexpression. (B) Representative images of subcutaneous tumors with control or *SLC2A11–MIF* overexpression are presented. (C) The weight (mg) of tumors with control or *SLC2A11–MIF* overexpression was measured after surgical dissection. (D) Representative images and a histogram of H&E and IHC staining images demonstrating Ki67 expression in tumors. Scale bars: 100 µm (black). (E) Representative images demonstrating tumor invasion into the surrounding muscle in the *SLC2A11–MIF*‐overexpressing group compared with the corresponding control group. The letter M denotes muscle, while T represents tumor. Arrows indicate areas of tissue invasion. (F) The histogram illustrates the percentage of tumor infiltration into the surrounding muscle between the *SLC2A11–MIF*‐overexpressing group and the control group. (G and H) Representative images of dissected popliteal lymph nodes and histogram analysis were performed to assess the volume of the lymph nodes. (I) LN status percentages in all groups (*n* = 6 per group). (J) Kaplan‒Meier survival analysis of mice inoculated with *SLC2A11–MIF*‐overexpressing or control cells. (K) Representative images of H&E and IHC staining were used to confirm the status of the lymph nodes (*n* = 6). Scale bars, 500 µm (black) and 50 µm (red). **p < 0.01.

To conduct a more in‐depth examination of the function of the S‐M‐S variant of *SLC2A11–MIF* in BCa, we conducted functional assays in BCa cells. CCK8, colony formation, and flow cytometry analyses revealed that *SLC2A11–MIF* overexpression considerably improved the proliferative capacity of BCa cells (Figures 4F–I and S4G). Additionally, we found that *SLC2A11–MIF* significantly increased the migration and invasion of BCa cells (Figures 4J–L and S4H). Subsequently, we administered stable *SLC2A11–MIF*‐overexpressing cells into the subcutaneous regions and footpads of nude mice. Intriguingly, the growth, tumor size, and weight of the *SLC2A11–MIF*‐overexpressing tumors were significantly increased (Figure 5A–C). Moreover, tumors derived from *SLC2A11–MIF*‐overexpressing cells exhibited upregulated Ki67 expression (Figure 5D) and invasive spike‐like structures that infiltrated the surrounding muscle tissues (Figure 5E,F). Furthermore, *SLC2A11–MIF*‐overexpressing mice exhibited a significant increase in popliteal lymph node volume, a decrease in survival rate, and a marked increase in the rate of lymphatic metastasis from 50 to 100% (Figure 5G–J). Additionally, the detection of metastatic lymph nodes was confirmed through H&E staining and luciferase immunohistochemistry (IHC) analysis (Figure 5K). Collectively, these findings provide compelling evidence supporting the role of *SLC2A11–MIF* in promoting tumor progression and metastasis in BCa cells in vitro and in vivo.

### 2.6 The fusion protein *SLC2A11–MIF* interacts with *PTBP1* to play an oncogenic role in BCa

**FIGURE 6:**
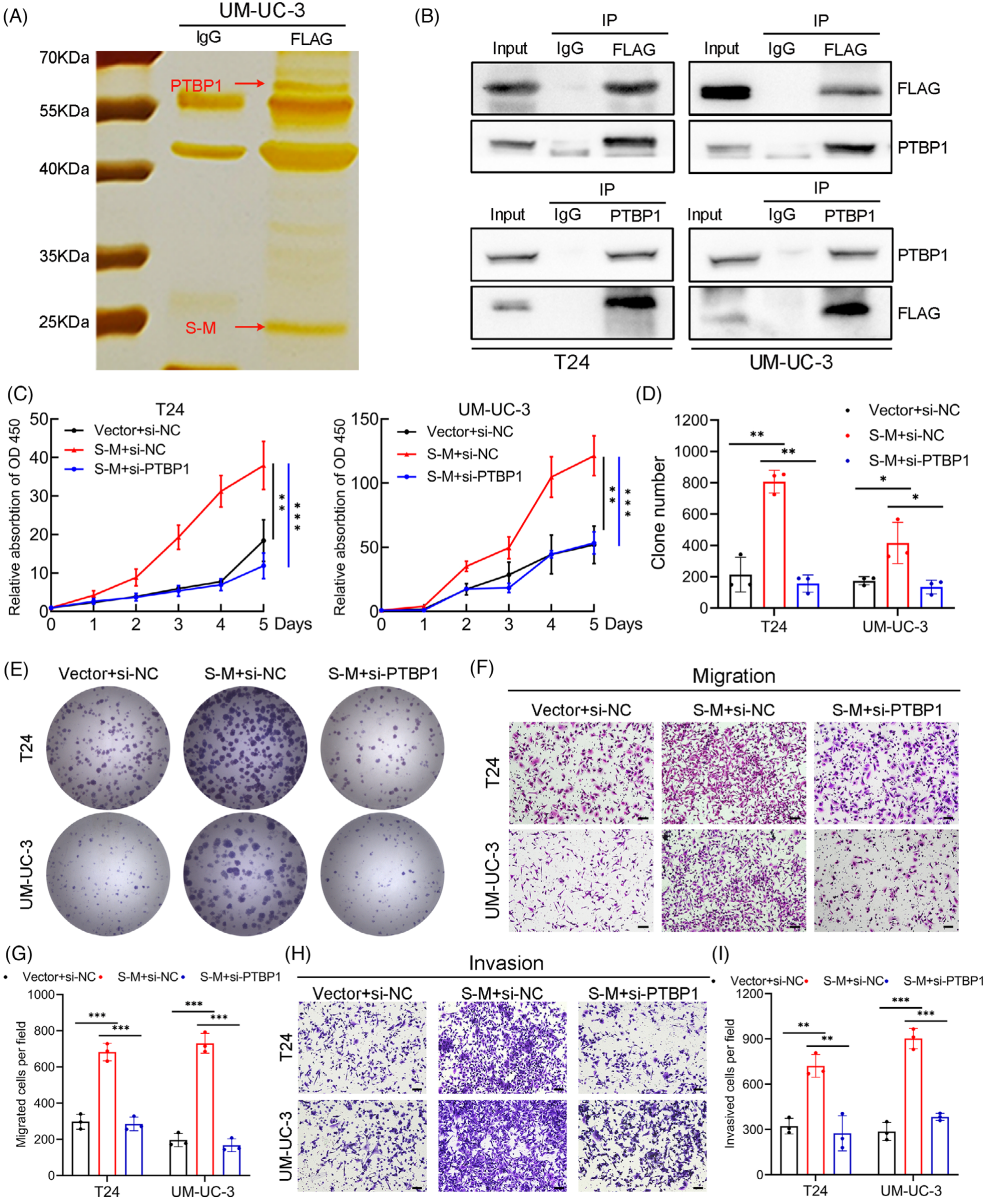
The fusion protein *SLC2A11–MIF* directly interacts with *PTBP1* to play key roles in BCa. (A) Co‐immunoprecipitation (Co‐IP) was conducted in S‐M‐S‐FLAG‐overexpressing T24 cells using an anti‐FLAG or negative control IgG antibody, followed by silver staining. The red arrows indicate the localization of *PTBP1* (above) and *SLC2A11–MIF* (below) protein bands. (B) Co‐IP and western blotting analysis revealed the interaction between *SLC2A11–MIF* and *PTBP1*. (C) Cell viability was evaluated in *SLC2A11–MIF*‐overexpressing or control cells with *PTBP1* knockdown in T24 and UM‐UC‐3 cells. (D and E) Colony formation was assessed in *SLC2A11–MIF*‐overexpressing or control cells in combination with *PTBP1* knockdown. (F and G) Representative images and histograms of migration were evaluated using *SLC2A11–MIF*‐overexpressing or control cells with *PTBP1* knockdown. Scale bars: black, 100 µm. (H and I) Representative images and histograms of invasion were evaluated using *SLC2A11–MIF*‐overexpressing or control cells with *PTBP1* knockdown. Scale bars: black, 100 µm. *p < 0.05, **p < 0.01, ***p < 0.001.

To elucidate the functional mechanism and identify protein interactors of the fusion protein *SLC2A11–MIF*, we conducted co‐immunoprecipitation (Co‐IP) followed by immunoprecipitation‐mass spectrometry. A distinct band ranging from 55 to 70 kDa was observed by silver staining and subsequently identified as *PTBP1* through mass spectrometry (Figures 6A and S5A). We further confirmed the specific interaction between *SLC2A11–MIF* and *PTBP1* using western blotting and immunofluorescence (Figures 6B and S5B). We previously demonstrated that *PTBP1* induces proliferation and lymphatic metastasis.^23^ To elucidate the functional significance of the *SLC2A11–MIF–PTBP1* complex, we overexpressed *SLC2A11–MIF* and subsequently knocked down *PTBP1* in the T24 and UM‐UC‐3 cell lines (Figure S5C). Importantly, depletion of *PTBP1* eliminated the *SLC2A11–MIF*‐induced increase in cell proliferation and metastasis in vitro (Figure 6C–I). Thus, these findings indicate that *SLC2A11–MIF* modulates proliferation and metastasis through a *PTBP1*‐dependent mechanism in bladder cancer cells.

### 2.7 *SLC2A11–MIF* modulates the stability of *PLK1*, *ROBO1*, and *PIK3R3* mRNA in a *PTBP1*‐mediated manner

**FIGURE 7:**
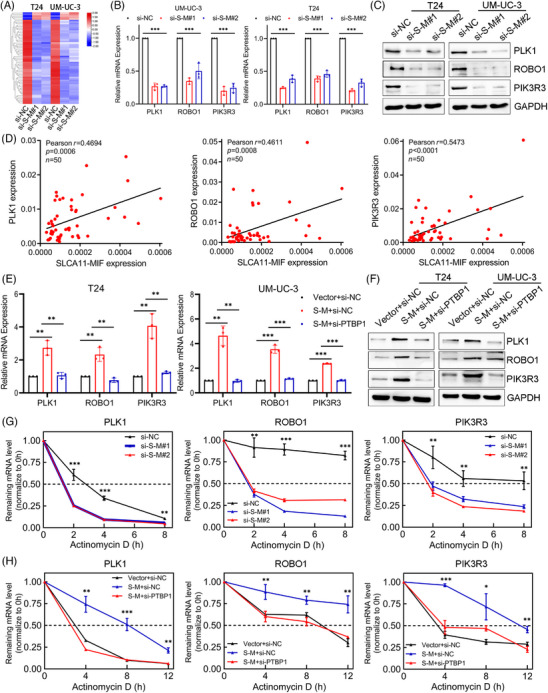
*SLC2A11–MIF* modulates the stability of the *PLK1*, *ROBO1*, and *PIK3R3* mRNAs in a *PTBP1*‐mediated manner. (A) A heatmap depicting mRNA expression in T24 and UM‐UC‐3 cells transfected with control or *SLC2A11–MIF* siRNA. (B) Differentially expressed genes identified from the microarray were validated by qRT‐PCR in T24 and UM‐UC‐3 cells. (C) Western blotting was performed to assess the expression of *SLC2A11–MIF* target genes, with *GAPDH* serving as the internal control. (D) Pearson correlations between the expression levels of *SLC2A11–MIF* and *PLK1*, *ROBO1*, and *PIK3R3* were determined by qRT‐PCR analysis of samples from 50 patients with bladder cancer. (E and F) mRNA and protein expression of *SLC2A11–MIF* target genes was evaluated in cells overexpressing *SLC2A11–MIF* or in control cells with *PTBP1* knockdown. (G) UM‐UC‐3 cells transfected with control or *SLC2A11–MIF* siRNA were treated with actinomycin D (5 mg/mL) for the indicated durations. (H) UM‐UC‐3 cells with stable control expression, *SLC2A11–MIF* overexpression, or *SLC2A11–MIF* overexpression with *PTBP1* siRNA were treated with actinomycin D (5 mg/mL) for the indicated time periods. Total RNA was purified and analyzed using qRT‐PCR to determine the mRNA half‐lives of *PLK1*, *ROBO1*, and *PIK3R3*. *p < 0.05, **p < 0.01, ***p < 0.001.

To clarify the molecular mechanism that underlies *SLC2A11–MIF*‐mediated proliferation and metastasis in BCa, we performed transcriptome sequencing to compare the gene expression profiles between *SLC2A11–MIF*‐silenced T24 and UM‐UC‐3 cells. Among the 61 genes subjected to regulatory control by *SLC2A11–MIF* (*p* value < 0.05, |log2FoldChange| > 1), we observed significant downregulation of several critical genes involved in cell proliferation and metastasis, including *PLK1*, *ROBO1*, and *PIK3R3*, upon silencing *SLC2A11–MIF* (Figures 7A and S6A–D). Furthermore, qRT‐PCR and western blotting revealed decreases in *PLK1*, *ROBO1*, and *PIK3R3* mRNA and protein expression in *SLC2A11–MIF*‐silenced cells (Figure 7B,C). Additionally, we detected a notable and favorable correlation between the expression of *SLC2A11–MIF* and the levels of *PLK1*, *ROBO1*, and *PIK3R3* in a cohort of BCa patients (Figure 7D). Considering the reported involvement of *PTBP1* in regulating mRNA stability,^24^ we investigated whether the fusion protein *SLC2A11–MIF* facilitates *PTBP1*‐mediated stabilization of target gene mRNAs. Interestingly, significant positive correlations were observed between *PTBP1* expression and *PLK1*, *ROBO1*, and *PIK3R3* expression in the TCGA cohort (Figure S6E). Additionally, we demonstrated that knockdown of *PTBP1* abrogated the *SLC2A11–MIF*‐mediated upregulation of target gene mRNA and protein expression (Figure 7E,F). Additionally, treatment with actinomycin D facilitated the quantification of preexisting mRNA decay. Our findings demonstrated that knockdown of *SLC2A11–MIF* decreased the half‐lives of the *PLK1*, *ROBO1*, and *PIK3R3* mRNAs (Figures 7G and S7A). Furthermore, *PTBP1* knockdown completely abrogated the *SLC2A11–MIF*‐induced increase in mRNA stability (Figures 7H and S7B). Collectively, these findings suggest that the interaction between *SLC2A11–MIF* and *PTBP1* plays a crucial role in stabilizing the mRNAs of *PLK1*, *ROBO1*, and *PIK3R3* and significantly contributes to their upregulation.

### 2.8 *SLC2A11–MIF* is a product of cis‐SAGe and its stability is mediated by *NAT10*

**FIGURE 8:**
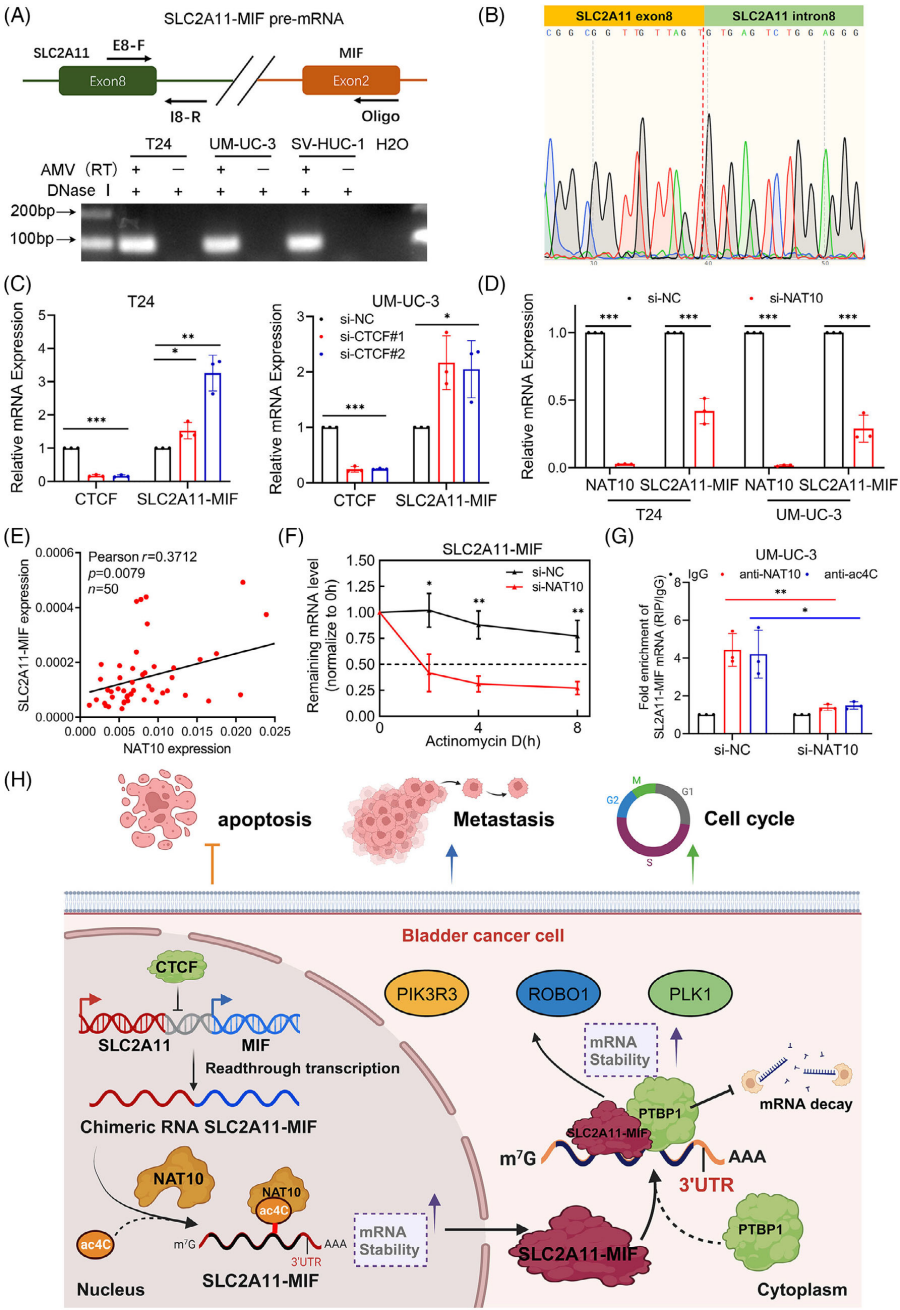
*SLC2A11–MIF* is a product of cis‐SAGe, and its stability is mediated by *NAT10*. (A) Top: Schematic showing cis‐splicing between adjacent genes (cis‐SAGe). Blocks represent exons, while lines represent introns or intergenic regions. The arrowhead indicates the oligo primer used for reverse transcription. The primers E8‐F and I8‐R anneal to exon 8 and intron 8 of *SLC2A11*, respectively. Bottom: RNA from the bladder cancer cell lines T24 and UM‐UC‐3, as well as from the human uroepithelial cell line SV‐HUC‐1, was initially treated with DNase I. Subsequently, the cells were divided into two groups: one with the avian myeloblastosis virus reverse transcriptase (AMV‐RT) enzyme and the other without it. The correct product was observed exclusively in samples containing the AMV‐RT enzyme. (B) Sanger sequencing confirmed the validity of the PCR products. (C) qRT‐PCR analysis was performed to evaluate the expression levels of *SLC2A11–MIF* in *CTCF*‐silenced cells and control cells. (D) The relative expression of *SLC2A11–MIF* was measured by qRT‐PCR after *NAT10* silencing. (E) Pearson correlations between the expression levels of *SLC2A11–MIF* and *NAT10* were determined by qRT‐PCR analysis using samples from 50 bladder cancer patients. (F) Change in *SLC2A11–MIF* mRNA stability was assessed by qRT‐PCR following actinomycin D treatment after *NAT10* knockdown. (G) RIP assays were conducted on UM‐UC‐3 cells to analyze the enrichment of *SLC2A11–MIF* mRNA relative to the nontargeting IgG control using qRT‐PCR analysis. (H) Proposed model of the interaction of the *SLC2A11–MIF* fusion protein with *PTBP1* to promote the proliferation and metastasis of bladder cancer cells via the regulation of mRNA stability. The image was created using BioRender.com. *p < 0.05, **p < 0.01, ***p < 0.001.

The *SLC2A11–MIF* fusion transcript is classified as a readthrough chimeric RNA due to the proximity of adjacent parental genes on the same chromosome.^2^, ^21^ Reverse transcription (RT) was performed using a downstream exon primer adjacent to the junction site, followed by RT‐PCR with primers covering a fragment of the 5′ gene. We detected signals only in the presence of the avian myeloblastosis virus RT (AMV‐RT) enzyme when primer pairs were used to amplify a cDNA fragment spanning exons 8 and intron 8 of *SLC2A11*, confirming that the precursor RNA was transcribed from exon 8 of *SLC2A11* to exon 2 of *MIF* (Figure 8A,B). *CTCF* plays a critical role in the binding process to insulator regions situated at or close to gene boundaries. Consequently, it inhibits cis‐splicing between two neighboring genes.^25^, ^26^ Interestingly, we observed significant upregulation of *SLC2A11–MIF* expression upon *CTCF* knockdown (Figure 8C), while the expression of the parental genes *SLC2A11* and *MIF* remained unaltered (Figure S8A). Hence, we can infer that the *SLC2A11–MIF* chimeric RNA is a product of cis‐SAGe.

Epi‐transcriptomic RNA modifications constitute a critical component of gene regulation that can impact cancer progression and metastasis. We investigated the relationship between *SLC2A11–MIF* and key RNA modifications derived from various RNA modification datasets. Notably, an online bioinformatics PACES database (http://www.rnanut.net/paces/) was used to predict the presence of a motif for N4‐acetylcytidine (ac4C) modification in *SLC2A11–MIF* (Figure S8B). A previous study demonstrated that *NAT10*‐mediated ac4C modification highly enriches mRNAs, enhances their stability, and promotes translational efficiency.^27^, ^28^ Intriguingly, knockdown of *NAT10* resulted in a significant decrease in the expression of *SLC2A11–MIF* mRNA (Figures 8D and S8C), while the expression levels of the parental genes *SLC2A11* and *MIF* remained unchanged (Figure S8D). Moreover, overexpression of *NAT10* led to upregulation of *SLC2A11–MIF* mRNA expression. (Figure S8E,F). Furthermore, a strong positive correlation was observed between *NAT10* and *SLC2A11–MIF* expression in a cohort of BCa patients (Figure 8E). Additionally, our findings suggest that *NAT10* plays a role in regulating the stability of *SLC2A11–MIF* mRNA following actinomycin D treatment (Figures 8F and S8G). Additionally, RNA immunoprecipitation (RIP) demonstrated substantial enrichment of *SLC2A11–MIF* mRNA through *NAT10*, which was abrogated upon silencing this protein. Furthermore, acRIP‐qPCR assays confirmed the reduced abundance of the ac4C modification on *SLC2A11–MIF* mRNA upon deletion of *NAT10* (Figure 8G). Therefore, we propose that *SLC2A11–MIF* represents a cis‐spliced product derived from adjacent genes and that its stability is regulated by *NAT10*.

## 3 DISCUSSION

Chimeric RNAs have been extensively detected in both normal human tissues and tumors.^29^, ^30^ Accumulating evidence suggests that chimeric RNAs play crucial roles in the initiation and progression of cancers.^20^, ^31^, ^32^ In previous investigations, ten highly prevalent chimeric RNAs were identified, among which *SLC2A11–MIF* exhibited a greater frequency of detection in bladder cancer tissues than previously reported gene fusions.^2^ In this study, we demonstrated that the overexpression of *SLC2A11–MIF* significantly augments the proliferation and metastasis of BCa cells. Mechanistically, the interaction between *SLC2A11–MIF* and *PTBP1* facilitates mRNA stabilization, thereby enhancing the expression of *PLK1*, *ROBO1*, and *PIK3R3*.

Chimeric RNAs have emerged as valuable biomarkers and promising therapeutic targets across various cancer types.^33^ Comprehensive analyses were also conducted to identify recurrent chimeric RNAs and explore their clinical implications in colon, cervical, and bladder cancers.^2^, ^21^, ^22^ The fusion of *SLC2A11–MIF* results in a truncated *SLC2A11* protein, which may have implications for the biology of both parental proteins. While *SLC2A11* plays a crucial role in glucose absorption and is commonly observed in cancer cells due to its high energy demand,^34^, ^35^, ^36^ and *MIF* is involved in cell proliferation, tumorigenesis, and metastasis across multiple cancers.^37^, ^38^, ^39^, ^40^ In this study, we demonstrated that *SLC2A11–MIF* promotes proliferation, metastasis, cell cycle progression and resistance to apoptosis in vitro and in vivo. Taken together, these findings contribute to a comprehensive understanding of the multifunctional role of *SLC2A11–MIF* in tumors for the first time and provide valuable insights for developing novel strategies for the early diagnosis and targeted treatment of BCa.

Chimeric RNAs can exert diverse effects on tumor progression through multifarious mechanisms, including their roles as long noncoding RNAs, fusion proteins and disruption of parental gene expression.^1^ Dysregulation of parental genes may play a role in tumorigenesis when a parental gene with pro‐oncogenic properties is combined with an enhanced promoter or when a fusion occurs between a parental gene that suppresses tumor growth and a sequence targeted by microRNA, as exemplified by *TMPRSS2–ERG*.^7^, ^19^ The fusion protein *EWSR1–ATF1* interacts with protein arginine methyltransferase 5 (*PRMT5*), thereby augmenting gene transcription for the maintenance of cell proliferation in clear cell sarcoma of soft tissue cells.^41^ In this study, we discovered a direct interaction between the fusion protein *SLC2A11–MIF* and *PTBP1*. The subcellular localization of *PTBP1* is crucial for its function, as cytoplasmic *PTBP1* interacts with target mRNAs to regulate splicing, polyadenylation, mRNA stability, and translation initiation.^42^, ^43^, ^44^, ^45^ A recent study reported that the long noncoding RNA *FIRRE* acts as a tumor promoter by interacting with *PTBP1* to stabilize *BECN1* mRNA and facilitate autophagy.^24^ We observed predominant colocalization of *SLC2A11–MIF* with *PTBP1* in the cytoplasm of BCa cells. *SLC2A11–MIF* modulates bladder cancer cell proliferation and metastasis through a *PTBP1*‐dependent mechanism. The interaction between *SLC2A11–MIF* and *PTBP1* plays a critical role in stabilizing the mRNAs of target genes.

In our previous study, silencing *SLC2A11–MIF* resulted in significant upregulation of *CDKN1A* in both HeLa and CaSki cells.^21^ Our findings unveil a novel mechanism through which *SLC2A11–MIF* facilitates *PTBP1*‐mediated stabilization of the *PLK1*, *PIK3R3*, and *ROBO1* mRNAs, thereby enhancing their expression levels. Importantly, all genes directly targeted by *SLC2A11–MIF* are implicated in multiple oncogenic events. Among these genes, *PLK1* is well known for its role in promoting cell cycle phase transitions.^46^, ^47^, ^48^, ^49^ Additionally, circular RNA_0001495 has been shown to enhance *ROBO1* expression and facilitate bladder cancer cell proliferation, migration, and invasion by sponging microRNA‐527.^50^ Furthermore, the overexpression of *PIK3R3* has been reported to be an oncogenic mechanism that promotes proliferation, metastasis, and resistance to apoptosis and chemotherapy in specific types of cancer.^51^, ^52^, ^53^, ^54^ Therefore, *SLC2A11–MIF* serves as an upstream regulator of these oncogenes involved in cell cycle progression, metastasis, and resistance to apoptosis. Targeting *SLC2A11–MIF* is a promising multipotent therapeutic strategy for impeding the proliferation and metastasis of patients with bladder cancer.

Cis‐SAGe is emerging as a promising strategy for generating chimeric fusion RNAs.^55^ A recent study revealed the induction of downstream gene transcription (DoGs) and their cis‐SAGe fusions under osmotic stress.^56^ A fast, easy, and versatile cell‐based reporter system was developed to identify regulators of cis‐SAGe.^57^ *CTCF* is a specific factor that binds to insulators between neighboring genes and has been demonstrated to impact certain cis‐SAGe chimeric RNAs.^25^, ^26^ In this study, the expression of *SLC2A11–MIF* significantly increased upon *CTCF* knockdown, indicating that cis‐SAGe is responsible for generating the *SLC2A11–MIF* chimeric RNA. However, the regulatory role of posttranscriptional RNA modifications in the expression of chimeric RNAs has not been determined. ac4C modification, catalyzed by *NAT10*, is widely observed in human mRNAs and helps stabilize mRNAs and enhance translation.^27^, ^58^ *NAT10* plays a pivotal role in bladder cancer cell lines by influencing proliferation, metastasis, and cisplatin chemoresistance.^28^, ^59^ Our study demonstrated that the downregulation of *NAT10* results in a decrease in the ac4C modification of *SLC2A11–MIF* mRNA, thereby compromising its stability and suggesting potential regulatory mechanisms for other chimeric RNAs.

In summary, we present a novel finding that *SLC2A11–MIF* plays a crucial role in the proliferation and metastasis of BCa through its ability to maintain mRNA stability (Figure 8H). Elucidating the precise involvement of *SLC2A11–MIF* in BCa progression will not only enhance our understanding of chimeric RNA‐mediated metastasis and proliferation but also facilitate the development of innovative therapeutic strategies for treating BCa metastasis.

## 4 MATERIALS AND METHODS

### 4.1 Patient tissue samples

This study utilized detection of the chimeric RNA *SLC2A11–MIF* in bladder cancer, along with normal tissue samples obtained from SYSMH. Sample collection was conducted in accordance with the guidelines outlined in the Declaration of Helsinki, and ethical approval was obtained from the Sun Yat‐Sen Memorial Hospital Ethics Committee. A total of 62 postoperative bladder cancer patients diagnosed pathologically and 32 normal tissue samples were selected from the SYSMH cohort for RNA extraction. These samples were collected between January 2010 and February 2023 from patients who underwent radical bladder cancer surgery at our hospital.

### 4.2 TCGA platform data mining

The TCGA database (https://cancergenome.nih.gov/) was utilized to investigate the clinical characteristics and prognosis of patients with bladder cancer and to evaluate the expression levels of *SLC2A11*, *MIF*, *PTBP1*, *PLK1*, *ROBO1*, and *PIK3R3*. The expression levels and correlation analyses of *PTBP1*, *PLK1*, *ROBO1*, and *PIK3R3* were obtained from GEPIA2 (http://gepia2.cancer‐pku.cn/#index) for bladder cancer, and Kaplan‒Meier survival analysis of *SLC2A11* and *MIF* in relation to patient prognosis was performed.

### 4.3 Cell culture

In this study, the human renal embryo cell line HEK‐293T and the human bladder cancer cell lines UM‐UC‐3 and TCCSUP were cultured in complete DMEM. The human bladder cancer cell lines T24 and 5637 were completely cultured with RPMI‐1640, and the human urothelial cell line SV‐HUC‐1 was completely cultured with Ham's F‐12K. The cell lines used in this study were obtained from the American Type Culture Collection (ATCC, Manassas, Virginia, USA). All cells were cultured according to established protocols.^60^ The cell lines were subjected to short tandem repeat analysis (IGE Biotechnology, Guangzhou, Guangdong, China) to ensure their authenticity and were confirmed to be free of mycoplasma contamination as well as cross‐contamination with other cell lines.

### 4.4 RNA interference

The siRNA oligonucleotide sequences used in this study were designed and synthesized by Gene Pharma (Shanghai, China). The knockdown efficiency of each siRNA on the target gene was verified through qPCR and Western blotting. The sequences of the siRNAs used in this study are shown in Table S1. Briefly, 200 µL of Opti‐MEM was added to a 1.5 mL EP tube, followed by the addition of 3 µL of Lipofectamine® RNAiMAX Reagent (Invitrogen, Carlsbad, California, USA), and 5 µL of siRNA was thoroughly mixed before incubation at 37°C overnight. Subsequently, the mixture was cultivated for an additional 24–48 h prior to subsequent experiments.^61^

### 4.5 IHC analysis

In this study, immunohistochemical staining was performed on the subcutaneous tumor tissue of mice and the foot pad tumor tissue of a mouse model with popliteal lymph node metastasis. IHC analysis was performed in accordance with a previously established protocol.^62^ The tissue scores were independently determined by two professional pathologists who were blinded to patient information. In cases where there was a significant disparity in the score results for the same tissue, rescoring was conducted until a consensus was reached. The specific scoring method employed was as follows: First, all sections were examined for staining intensity and categorized into four levels. The percentage of positively stained tumor cells was scored as follows: 0 (no positive staining), 1 (≤10% positive), 2 (>10% to ≤30% positive), 3 (>30% to ≤70% positive), or 4 (>70% positive). The staining intensity was graded as follows: 1 (no staining), 2 (weak staining, light yellow), 3 (moderate staining, brown), or 4 (strong staining, brown red). The staining index (SI) was calculated by multiplying the proportion of positively stained tumor cells by the corresponding intensity score for a range of possible scores, which included 0, 1, 2, 3, 4, 6, 8, 9, 12, and 16.

### 4.6 RNA isolation and qPCR

RNA isolation and qRT‐PCR were conducted according to previously established protocols.^63^ In this study, TRIzol (Vazyme, Nanjing, Jiangsu, China) was used for the extraction of cellular RNA. The RT procedure was conducted following the instructions provided in the HiScript III RT SuperMix for qPCR manual (Vazyme). Quantitative PCR analysis was performed using ChamQ Universal SYBR qPCR Master Mix (Vazyme) on a LightCycler 384 System (Roche). Relative mRNA expression levels of different genes were calculated by the 2^−ΔΔCt^ method with GAPDH as an internal reference. The sequences of primers used for analysis are presented in Table S2.

### 4.7 Western blotting

The Western blotting experiments were performed in accordance with previously established protocols.^60^ The cell samples were lysed using RIPA lysis buffer (Beyotime, Shanghai, China) containing protease inhibitors and phosphatase inhibitors (CWBIO, Beijing, China) for 30 min. The protein concentration was determined using the Pierce BCA Protein Assay Kit (Invitrogen, Carlsbad, California, USA). Following SDS‒PAGE, the proteins were transferred to PVDF membranes (Merck, Burlington, Massachusetts, USA) by electrophoretic transfer. The membranes were incubated with primary antibodies overnight at 4°C. Subsequently, the membranes were incubated with an HRP‐conjugated secondary antibody for 1 h, and the signals were detected using a Sage high‐sensitivity chemiluminescence detector with a supersensitive luminescent solution. Primary antibodies are listed in Table S3.

### 4.8 Cell proliferation assays

Cell proliferation assays included CCK‐8 and colony formation assays in accordance with previously established protocols.^64^ The incubation with CCK‐8 (APExBIO, USA) for 2 h was assessed by quantifying the optical density (OD) at a wavelength of 450 nm.

### 4.9 Flow cytometry analysis

An Annexin V‐FITC/PI Apoptosis Detection Kit (A211‐01; Vazyme) was used to detect cell apoptosis. Additionally, the Cell Cycle Detection Kit (KGA512; KeyGEN, Jiangsu, China) was utilized to assess the distribution of different cell cycle stages during cellular growth according to the respective protocols. The cell cycle distribution was assessed using flow cytometry (BD Biosciences, San Jose, California, USA) with propidium iodide (PI) labeling. Additionally, FITC‐annexin V and PI staining were employed for the apoptosis assay.

### 4.10 Wound healing, migration, and invasion assays

Wound healing, migration, and invasion assays were conducted in accordance with our previous study.^65^

For wound healing assays, once the bladder cancer cells had completely covered the single hole of the six‐well plate, a sterile pipette tip was used to create a uniform horizontal mark at the center of the hole. Subsequently, the floating cells were washed away using PBS and maintained in serum‐free medium. The wound healing widths were measured at 0 and 16 h for T24 cells and UM‐UC‐3 cells, depending on their motility. The relative migratory ability of the treated cells was assessed based on the recorded wound healing width.

For migration assays, 8 × 10^4^ BCa cells were seeded onto the upper layer of a Transwell plate with a pore size of 8 µm, while the lower chamber was filled with 700 µL of complete medium. The incubation period for the T24 cells was 7.5 h, whereas the UM‐UC‐3 cells were incubated for 20 h. Following fixation with 4% paraformaldehyde, crystal violet staining was performed, and subsequent quantification was conducted.

For invasion assays, the upper chamber of a Transwell plate with an 8 µm pore size was coated with Matrigel (Corning, Bedford, Massachusetts, USA) and allowed to solidify. Subsequently, 8 × 10^4^ BCa cells were seeded onto the upper layer of the Transwell plate, while the lower chamber was filled with complete medium. T24 cells were incubated for 10 h, whereas UM‐UC‐3 cells were incubated for 24 h. After fixation with 4% paraformaldehyde, crystal violet staining was performed, followed by quantification.

### 4.11 Tumor xenograft model

All animal studies were ethically approved by the Institutional Animal Care and Use Committee of Sun Yat‐Sen University. Male BALB/c nude mice (4–5 weeks old) were procured from the Experimental Animal Center of Sun Yat‐Sen University and housed in specific pathogen‐free barrier facilities. A total of 3 × 10^6^ UM‐UC‐3 cells were subcutaneously injected beneath the right scapula of nude mice. The injection site was monitored daily postinjection, and the size of the subcutaneous tumor was measured and recorded every 3 days. The experimental endpoint criteria were met by employing humane euthanasia followed by cervical dislocation to sacrifice the mice. Subsequently, the dissected subcutaneous tumors were photographed, measured, weighed, and fixed with 4% paraformaldehyde. After fixation, paraffin embedding was performed, and the largest section was selected for further processing. Histological analysis, including H&E staining, Ki67 immunostaining, and TUNEL assays, was conducted on these sections.

### 4.12 In vivo popliteal LN metastasis assays

UM‐UC‐3 cells transfected with firefly luciferase (3 × 10^6^ cells) were injected into the left foot pads of the mice. Once tumor formation was observed in situ, in vivo imaging analysis of the nude mice was performed every 7 days using a PerkinElmer IVIS Spectrum Imaging System. The experimental endpoints were determined based on the in vivo imaging results and ethical considerations. Euthanasia was conducted by cervical dislocation. The foot pad tumor and popliteal fossa lymph nodes were dissected for analysis, measurement, fixation with paraformaldehyde, embedding in paraffin wax, and sectioning to obtain the largest slice, followed by H&E staining and IHC. Survival curves and transfer rates in the mice were calculated.

### 4.13 TUNEL assay

The TUNEL assay was performed using a TUNEL FITC Apoptosis Detection Kit (A111‐01; Vazyme) following a previously established protocol.^66^

### 4.14 Lentivirus transduction

The pLKO.1‐Puro vector was utilized to clone and insert two short hairpin RNA (shRNA) sequences specifically targeting *SLC2A11–MIF*, while the pCDH–CMV–MCS–EF1–Puro vector was used for cloning and insertion of the ORF of *SLC2A11–MIF*. Both vectors were obtained from IGE (Guangzhou, Guangdong, China). Table S1 provides a comprehensive list of all shRNA sequences used in this study. Lentivirus production and infection were conducted following established protocols.^49^

### 4.15 RNA sequencing analysis

The cells were transfected with si‐*SLC2A11–MIF* or control siRNA for 48 h. Subsequently, total RNA was extracted from the cells using TRIzol (Vazyme) and subjected to RNA‐Seq analysis. The sequencing library was constructed and sequenced by IGE Biotechnology (Guangzhou, China). All primary data in the RNA‐seq analysis were uploaded to Gene Expression Omnibus (GEO: GSE271133, https://www.ncbi.nlm.nih.gov/geo/query/acc.cgi?acc=GSE271133).

### 4.16 Coimmunoprecipitation

A Pierce Crosslink Magnetic Co‐IP Kit (Thermo Scientific, USA) was used for Co‐IP following the manufacturer's instructions and our previous study.^60^ Briefly, lysed cells were incubated in IP lysis buffer containing a protease inhibitor mixture. Subsequently, they were incubated with anti‐FLAG, anti‐PTBP1, or IgG antibodies. Antibodies for Co‐IP (as listed in Table S3) and cell lysates were rotated at 4°C overnight. A&G magnetic beads were then added and rotated for 2 h. The resulting bead‐protein complexes were washed three times and boiled for 10 min at 95°C before Western blot analysis. MS analysis was conducted by the Bioinformatics and Omics Center of Sun Yat‐Sen Memorial Hospital.

### 4.17 Immunofluorescence staining

Immunofluorescence staining was performed according to established protocols.^28^ The cells were fixed using a 4% paraformaldehyde solution, followed by permeabilization with 0.2% Triton X‐100. Subsequently, the membranes were incubated with the primary antibody overnight at 4°C and then blocked with a BSA solution. Finally, the cells were incubated with the secondary antibody and DAPI before the images were captured.

### 4.18 Detection of precursor readthrough mRNA

DNase‐I was applied to eliminate potential DNA contamination in RNA isolated from the bladder cancer cell lines T24 and UM‐UC‐3 and the normal uroepithelial cell line SV‐HUC1. RT was performed using reverse primer annealing to the downstream exon neighboring the junction site, followed by RT‐PCR with primers covering a fragment of the 5′ gene. Complete digestion by DNase‐I was confirmed through the absence of a signal without AMV‐RT reverse transcriptase.

### 4.19 Detection of full‐length *SLC2A11–MIF*

Initially, we trimmed the junction to 14 base pairs on both sides and included the reverse complements. Subsequently, using AGREP, we conducted a comprehensive search for matches of each sequence (allowing 0 or 2 errors) in all the ENCODE samples. The matched reads were subsequently subjected to BLAT and a blat filter called reps for optimal alignment identification with the genome. Finally, we compared the alignment coordinates to a significantly large (∼300 kb) region surrounding the predicted locus of the chimeric RNA.

### 4.20 Statistics

The quantitative data are presented as the mean ± standard deviation (SD) of three independent experiments. Statistical analysis was performed using SPSS 19.0 software, and differences between two groups were assessed using unpaired/paired Student's *t*‐tests (two‐tailed tests). For comparisons involving more than two groups, one‐way ANOVA followed by Dunnett's multiple comparisons test was used. Clinical variables were analyzed using Pearson's chi‐square test, while Spearman's correlation analysis was used to determine the relationship between two variables. A *p* value less than 0.05 was considered to indicate statistical significance.

## AUTHOR CONTRIBUTIONS

X. C., H. L., and J. H. designed the study. L. C., C. W. Y., and J. L. L. conducted the main experiments and performed the data analysis. M. H. and R. H. X. analyzed the clinical characteristics. Sarah L. and J. E. performed the bioinformatic analysis. Sen L., Y. H. H., and S. T. C. conducted the in vitro and in vivo functional experiments. B. Q. H. and T. X. L. conducted the statistical analyses. X. C., H. L., and J. H. wrote and reviewed the manuscript. All the authors read and approved the final manuscript. The order of authorship among co‐first authors was determined based on their relative contributions.

## CONFLICT OF INTEREST STATEMENT

The authors declare no conflict of interest.

## ETHICS STATEMENT

The ethical consent of this study was obtained from Sun Yat‐Sen Memorial Hospital Committees for Ethical Review of Research involving Human Subjects (SYSKY‐2022‐392‐0). All human tissue samples were obtained from patients who provided written informed consent. Ethical approval for this study was granted by the Sun Yat‐Sen University Committees for Ethical Review of Research Involving Animal Experiments (SYSU‐IACUC‐2021‐000399).

## Supporting information

Supporting Information

## Data Availability

The descriptions of data mining in the TCGA study are provided in *Materials and Methods* section. The raw RNA‐seq data can be obtained from the GEO database (GSE271133, https://www.ncbi.nlm.nih.gov/geo/query/acc.cgi?acc=GSE271133).
