# Supplementary material for: Oncogenic SLC2A11–MIF fusion protein interacts with polypyrimidine tract binding protein 1 to facilitate bladder cancer proliferation and metastasis by regulating mRNA stability
Source: MedComm (2020). 2024 Aug 14;5(9):e685. doi: 10.1002/mco2.685 (PMC11324686; doi:10.1002/mco2.685)
Supplement: Supplementary file 1 — Supporting Information [file MCO2-5-e685-s001.docx]

**Oncogenic *SLC2A11-MIF* Fusion Protein Interacts with Polypyrimidine Tract Binding Protein 1 to Facilitate Bladder Cancer Proliferation and Metastasis by Regulating mRNA Stability**

**Running Title: *SLC2A11-MIF* Promotes Metastasis in Bladder Cancer**

Liang Cheng 1,2, #, Chenwei Yang1,2, #, Junlin Lu1,2, #, Ming Huang1,2,3, Ruihui Xie1,2,3, Sarah Lynch4, Justin Elfman4, Yuhang Huang1,2, Sen Liu1,2, Siting Chen1,2, Baoqing He1,2, Tianxin Lin 1,2,3, Hui Li4*, Xu Chen1,2,3,* & Jian Huang 1,2,3*.

1 Department of Urology, Sun Yat-sen Memorial Hospital, Sun Yat-sen University, Guangzhou, Guangdong, P. R. China.

2 Guangdong Provincial Key Laboratory of Malignant Tumor Epigenetics and Gene Regulation, Sun Yat-sen Memorial Hospital, State Key Laboratory of Oncology in South China, Guangzhou, Guangdong, P. R. China.

3 Guangdong Provincial Clinical Research Center for Urological Diseases, Guangzhou, Guangdong, P. R. China.

4 Department of Pathology, School of Medicine, University of Virginia, Charlottesville, VA 22908, USA.

# Liang Cheng, Chenwei Yang, and Junlin Lu contributed equally to this work.

***Corresponding to:**

Jian Huang, Department of Urology, Sun Yat-sen Memorial Hospital, 107th Yanjiangxi Road, Guangzhou, China, Tel: 86-1360004833; Fax: 86-20-34070392, E-mail: [huangj8@mail.sysu.edu.cn](mailto:huangj8@mail.sysu.edu.cn); Xu Chen, E-mail: [chenx457@mail.sysu.edu.cn](mailto:chenx457@mail.sysu.edu.cn); Hui Li, E-mail: hl9r@virginia.edu.


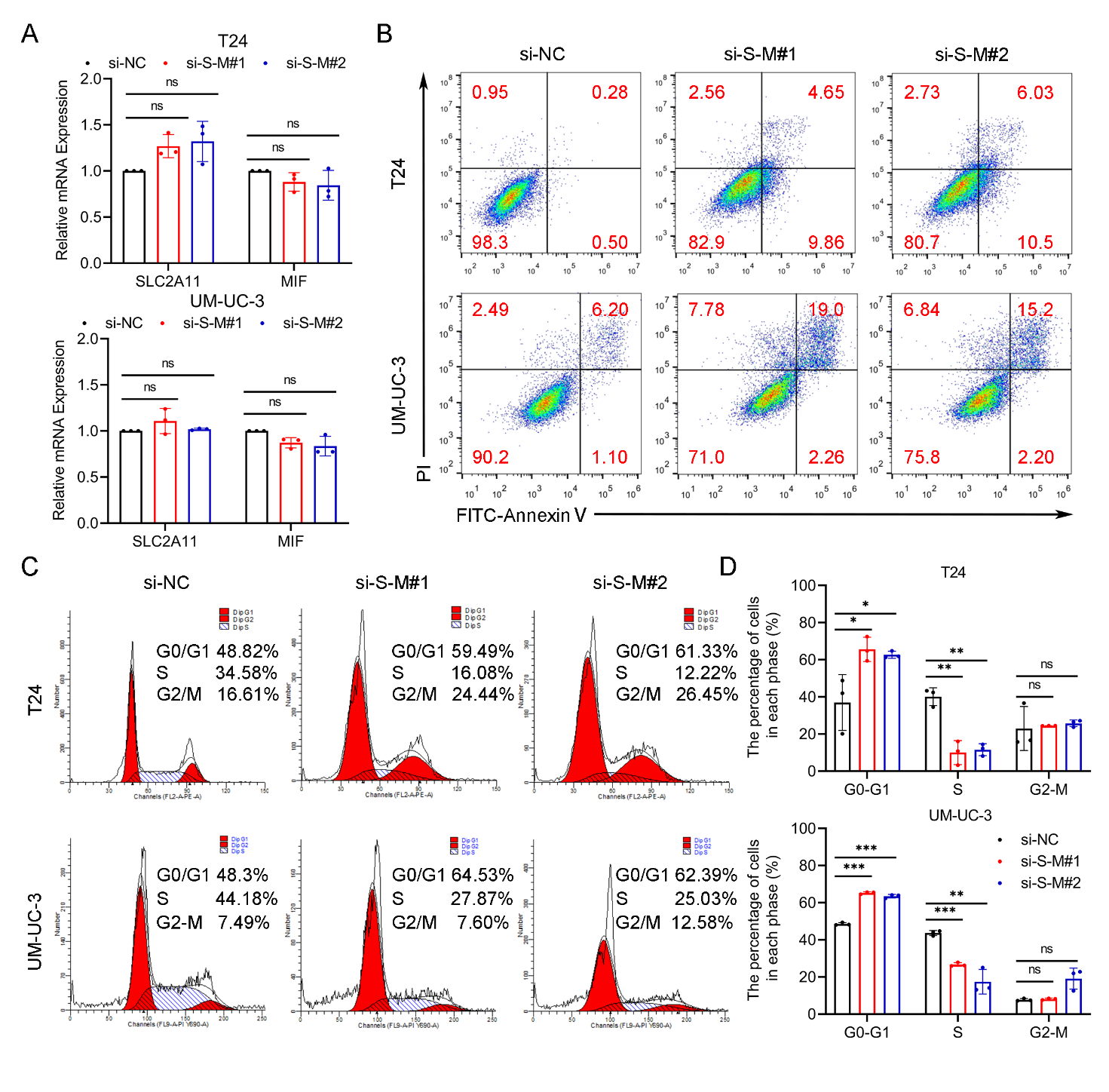
**Supplementary figure legends**

Figure S1. **Knockdown of *SLC2A11-MIF* inhibited proliferation *in vitro.***

(A) qRT‒PCR analysis was conducted to assess the expression of *SLC2A11* and *MIF* in BCa cells with silenced *SLC2A11-MIF* and control cells. (B) Images of cell apoptosis were obtained from T24 and UM-UC-3 cells transfected with control or *SLC2A11-MIF* siRNAs. (C-D) Flow cytometry analysis was conducted on T24 and UM-UC-3 cells transfected with *SLC2A11-MIF* siRNAs compared to the corresponding control cells. The percentages (%) of cell populations at different stages of the cell cycle are provided. **p* < 0.05, ***p* < 0.01, ****p* < 0.001, ns indicates not statistically significant.


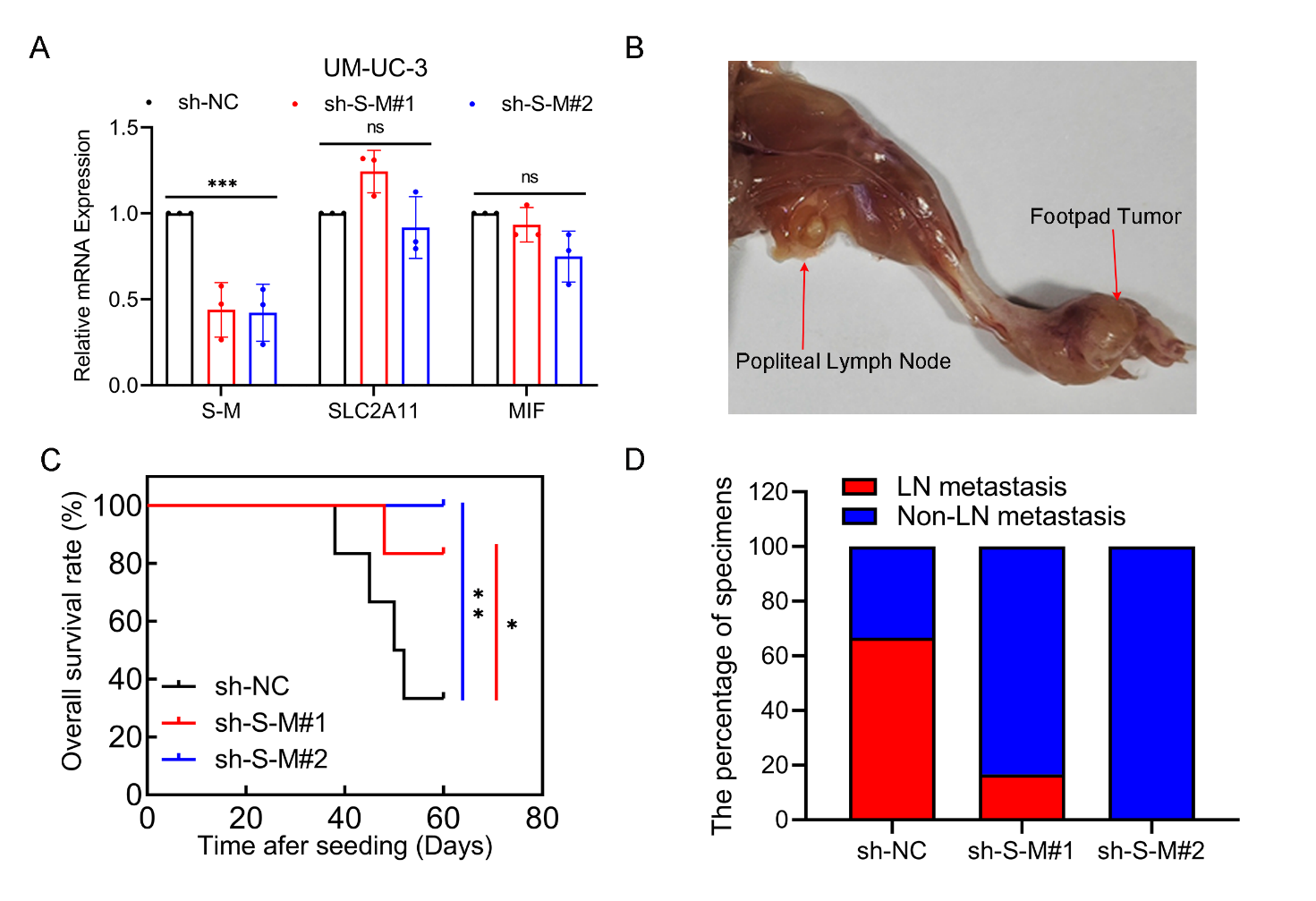
Figure S2. **Knockdown of *SLC2A11-MIF* suppressed LN metastasis *in vivo***

(A) Expression levels of *SLC2A11-MIF*, *SLC2A11*, and *MIF* were analysed using qRT-PCR in sh-NC, sh-S-M#1, and sh-S-M#2 samples. (B) Representative images of the nude mouse model of popliteal LN metastasis were obtained. UM-UC-3 cells were injected into the footpads of nude mice, and the popliteal LNs were surgically removed for analysis. (C) Kaplan-Meier survival analysis of mice inoculated with *SLC2A11-MIF*-knockdown or control UM-UC-3 cells. (D) LN status percentages in all groups (n = 6 per group). **p* < 0.05, ***p* < 0.01, ****p* < 0.001, ns indicates not statistically significant.


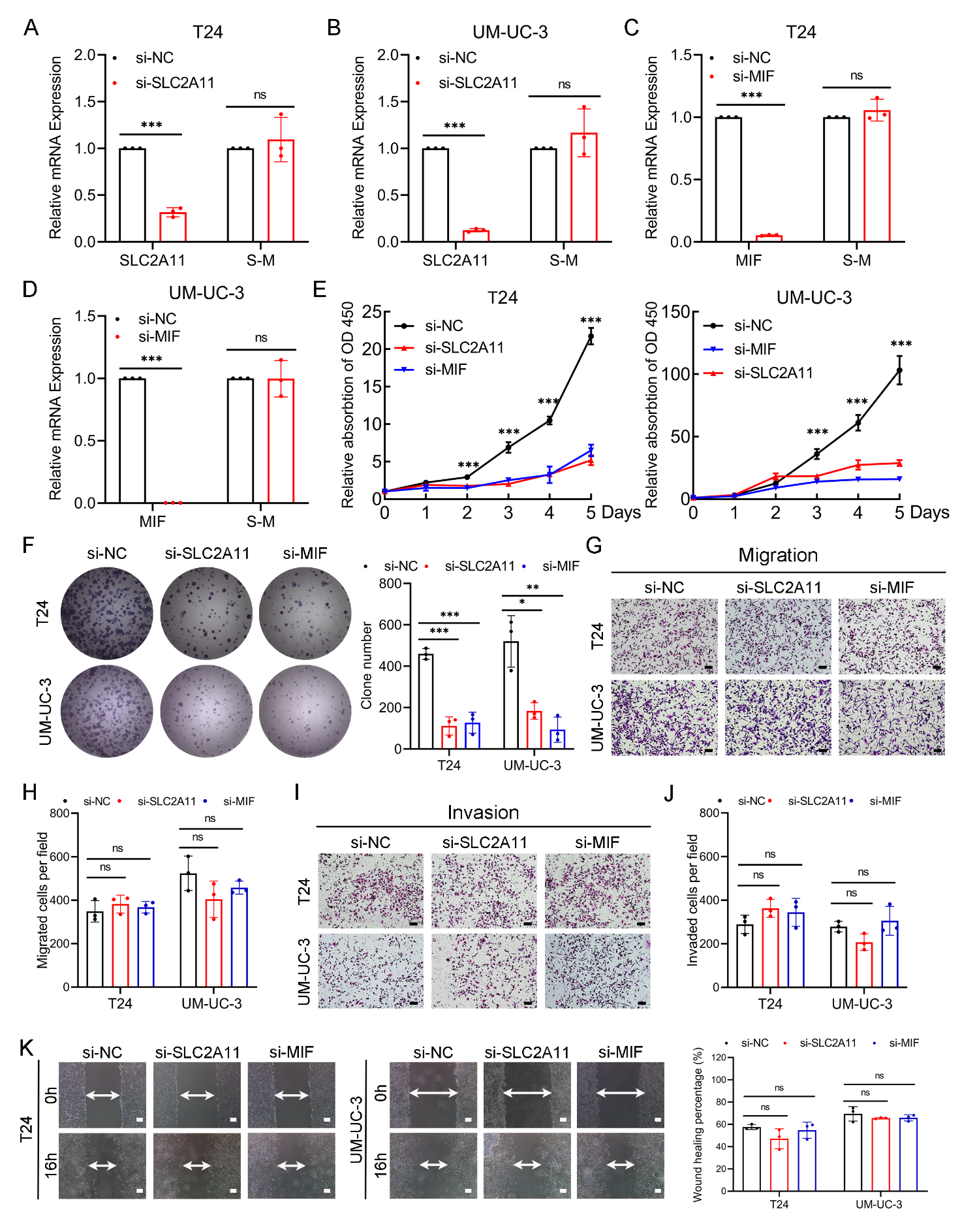
Figure S3. **Knockdown of parental genes inhibited proliferation but not metastasis *in vitro.***

(A-B) qRT‒PCR analysis was conducted to assess the expression of *SLC2A11* and *SLC2A11-MIF* in BCa cells with silenced *SLC2A11* and control cells. (C-D) qRT‒PCR analysis was conducted to assess the expression of *MIF* and *SLC2A11-MIF* in BCa cells with silenced *MIF* and control cells. (E) Cell viability was evaluated in T24 and UM-UC-3 cells with knockdown of *SLC2A11* or *MIF*. (F) Colony formation assays were performed in T24 and UM-UC-3 cells with knockdown of *SLC2A11* or *MIF*. (G-H) Representative images and histograms of migration assays in T24 and UM-UC-3 cells reveal decreased cell migratory capacity after knockdown of *SLC2A11* or *MIF*. (I-J) Representative images and histograms illustrating the invasion of T24 and UM-UC-3 cells after silencing *SLC2A11* or *MIF*. (K) Representative images and histograms from wound healing assays demonstrating cellular motility following knockdown of *SLC2A11* or *MIF*. **p* < 0.05, ***p* < 0.01, ****p* < 0.001, ns indicates not statistically significant.


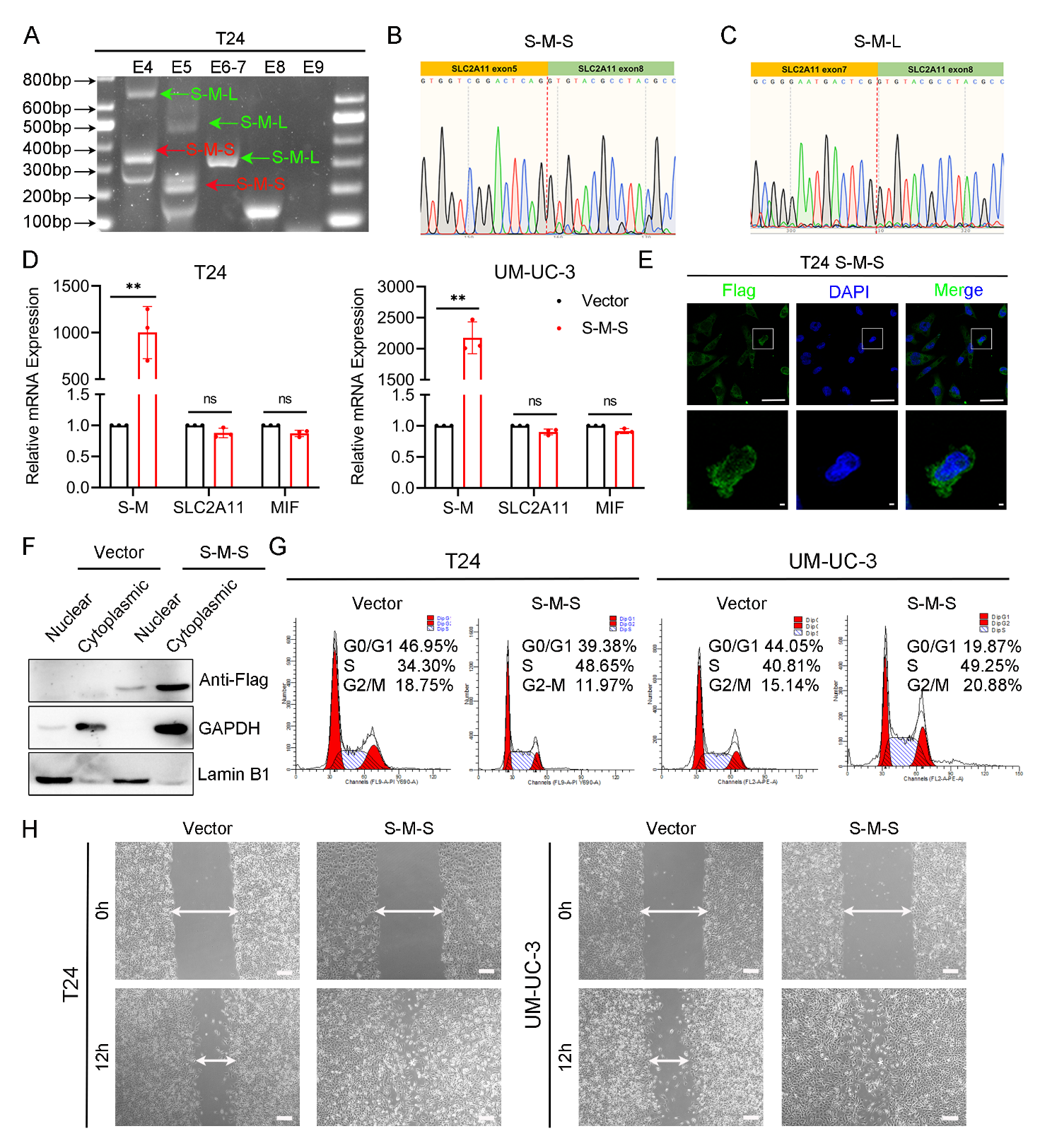
Figure S4. **Characterization and functional analysis of the fusion protein *SLC2A11-MIF*.**

(A) The expression levels of the S-M-L and S-M-S isoforms in T24 cells were evaluated by RT-PCR. (B-C) The validity of the PCR products for S-M-S and S-M-L was confirmed through Sanger sequencing. (D) qRT-PCR analysis was conducted to assess the expression levels of *SLC2A11-MIF*, *SLC2A11*, and *MIF* in cells transfected with *SLC2A11-MIF*-overexpression and control plasmids. (E) The subcellular distribution of *SLC2A11-MIF*-FLAG was visualized by immunofluorescence in T24 cells with DAPI staining of the nucleus. Scale bars, 50 μm (white). (F) Nuclear fractionation followed by western blotting detected *SLC2A11-MIF*-FLAG abundance in both the nuclear and cytoplasmic compartments, with GAPDH and Lamin B1 serving as internal controls for cytoplasmic and nuclear fraction separation, respectively. (G) Representative images depicting cell cycle progression in T24 and UM-UC-3 cells overexpressing *SLC2A11-MIF*. (H) Representative images from wound healing assays demonstrating cellular motility following overexpression of *SLC2A11-MIF* in T24 and UM-UC-3 cells. ***p* < 0.01, ns indicates not statistically significant.


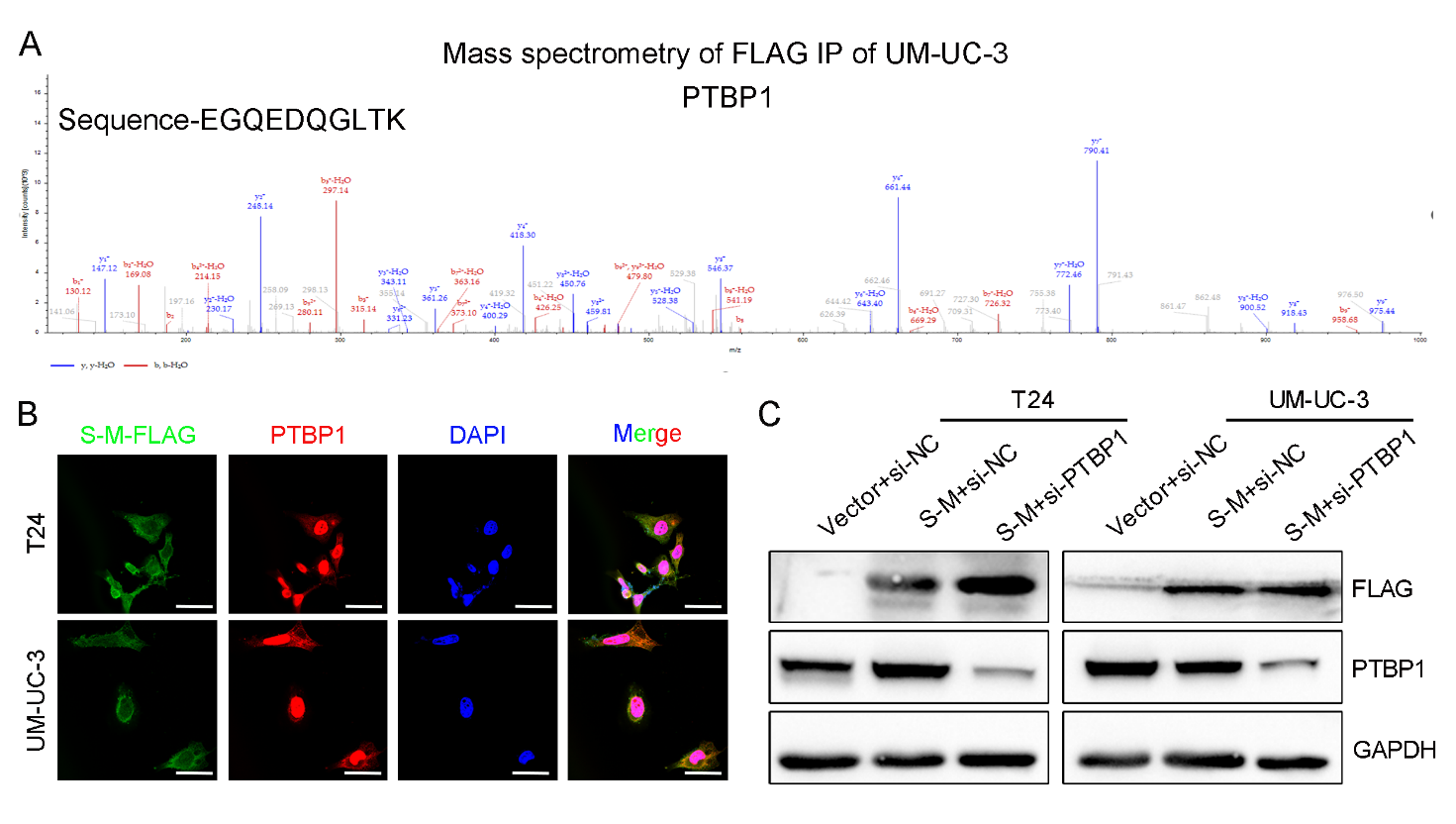
Figure S5. **The fusion protein *SLC2A11-MIF* directly interacts with *PTBP1* to play key roles in BCa.**

(A) IP-MS identification of the *SLC2A11-MIF*-interacting protein *PTBP1* in UM-UC-3 cells. (B) Representative IF images demonstrating the colocalization of *SLC2A11-MIF*-FLAG and *PTBP1* in the cytoplasm of bladder cancer cells. Blue represents nuclei, green represents FLAG, and red represents *PTBP1*. Scale bars: white, 25 μm. (C) Western blot analysis of *SLC2A11-MIF*-FLAG and *PTBP1* expression in *SLC2A11-MIF*-overexpressing or control BCa cells with *PTBP1* knockdown.


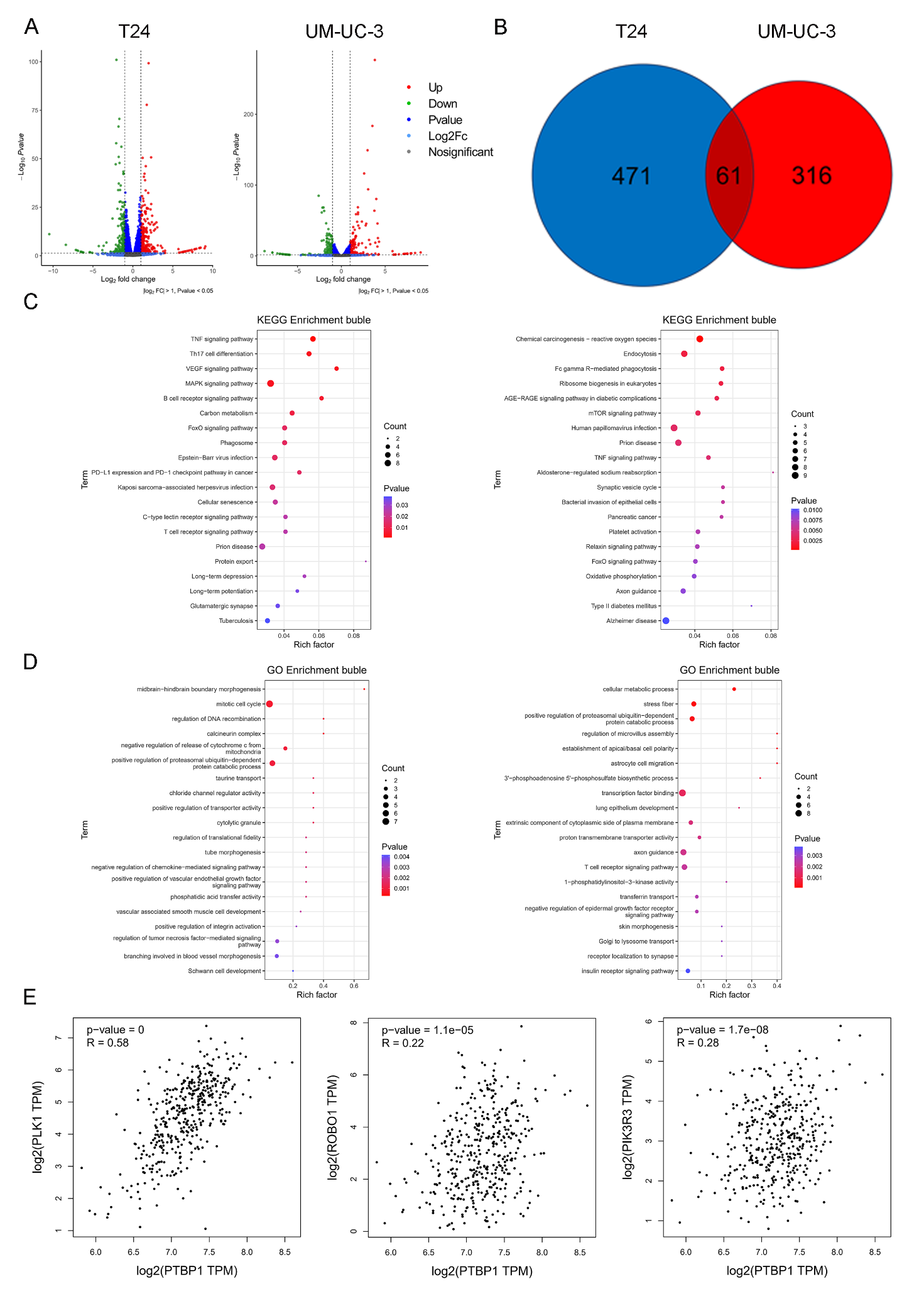
Figure S6. ***SLC2A11-MIF* modulates the stability of *PLK1*, *ROBO1* and *PIK3R3* mRNAs in a *PTBP1*-mediated manner.**

(A) Volcano plot illustrating the differential expression of genes in T24 and UM-UC-3 cells following *SLC2A11-MIF* knockdown for 48 hours, as determined by RNA sequencing. (B) Venn diagram demonstrating the number of downregulated genes specifically observed in T24 (left) and UM-UC-3 (right) cells upon knockdown of *SLC2A11-MI*F. (C) Kyoto Encyclopedia of Genes and Genomes (KEGG) analysis identifying the enrichment of biological processes in T24 and UM-UC-3 cells following *SLC2A11-MIF* knockdown. (D) Gene Ontology (GO) analysis identifying the enrichment of biological processes in T24 and UM-UC-3 cells following *SLC2A11-MIF knockdown*. (E) Pearson correlations between the expression of *PTBP1* and that of *PLK1*, *ROBO1* and *PIK3R3* in the TCGA BLCA cohort.


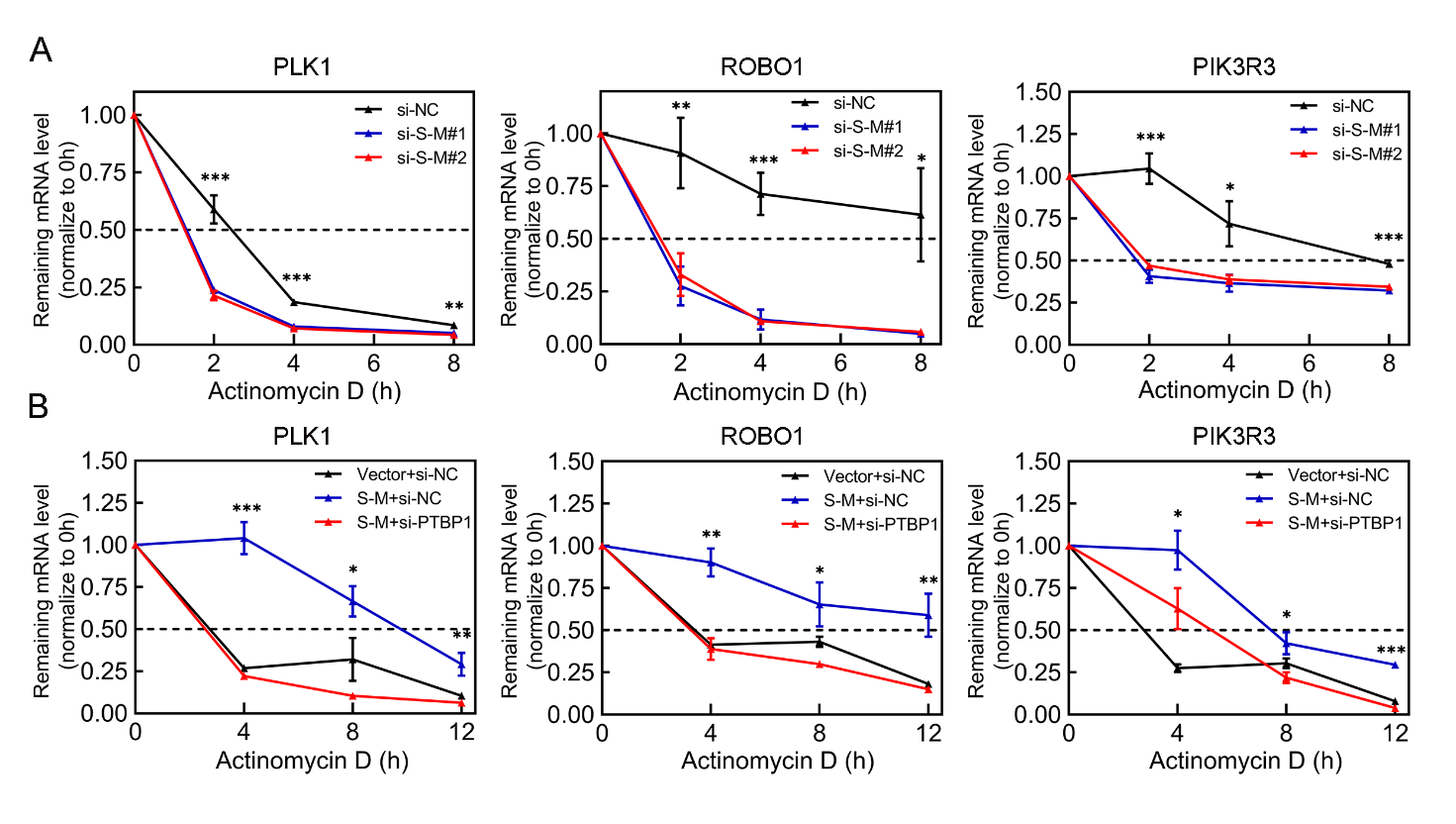
Figure S7 ***SLC2A11-MIF* modulates the stability of *PLK1*, *ROBO1* and *PIK3R3* mRNAs in a *PTBP1*-mediated manner in T24.**

(A) T24 cells transfected with control siRNA or *SLC2A11-MIF* siRNA were treated with actinomycin D (5 mg/ml) for the indicated durations. (B) T24 cells with stable expression of control, *SLC2A11-MIF*-overexpressing, or *SLC2A11-MIF*-overexpressing with *PTBP1* siRNA were treated with actinomycin D (5 mg/ml) for the indicated time periods. Total RNA was purified and analysed using qRT‒PCR to determine the mRNA half-lives of *PLK1*, *ROBO1*, and *PIK3R3*. **p* < 0.05, ***p* < 0.01, ****p* < 0.001.


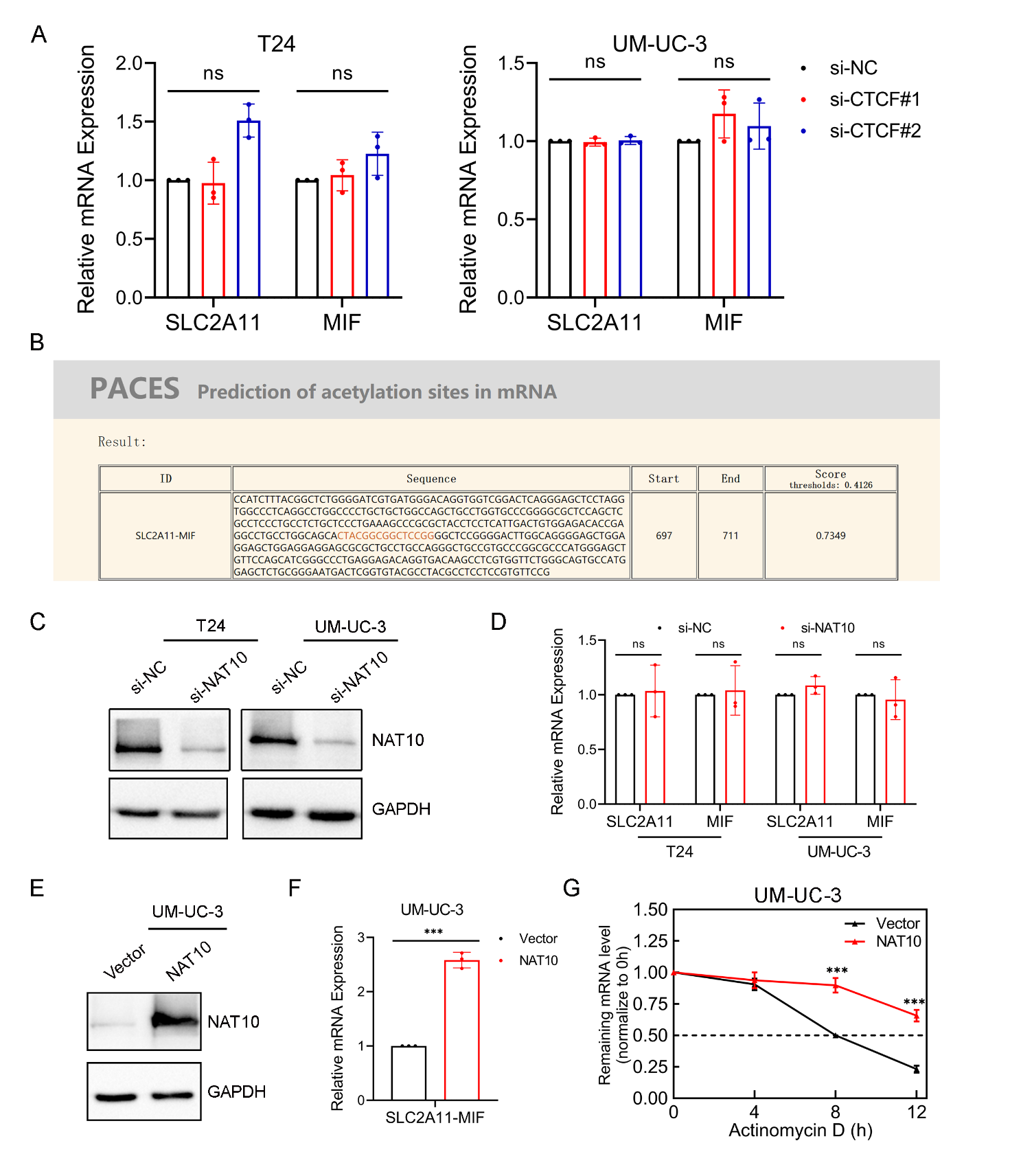
Figure S8. ***SLC2A11-MIF* is regulated by *CTCF* and *NAT10***

(A) qRT‒PCR analysis was performed to evaluate the expression levels of *SLC2A11* and *MIF* in *CTCF*-silenced cells and control cells. (B) The presence of the ac4C consensus motif in *SLC2A11-MIF* was investigated in the PACES database. (C) Western blot analysis was performed to evaluate the expression levels of *NAT10* in si-NC and si-*NAT10*-transfected BCa cells. (D) qRT‒PCR analysis was conducted to assess the expression of *SLC2A11* and *MIF* in BCa cells with silenced *NAT10* and control cells. (E) Western blotting was carried out to assess the expression of *NAT10* in *NAT10*-overexpressing cells and control cells, using *GAPDH* as an internal control. (F) The relative expression of *SLC2A11-MIF* was measured by qRT–PCR after overexpression of *NAT10*. (G) Changes in the mRNA stability of *SLC2A11-MIF* were assessed by qRT‒PCR following treatment with actinomycin D after overexpression of *NAT10*. ****p* < 0.001, ns indicates not statistically significant.

**Supplementary Table S1. Sequences of siRNA oligos and shRNAs**

| **Name** | **Sequence 5’-3’** |
| --- | --- |
| **siRNA** |  |
| si-NC | UUCUCCGAACGUGUCACGUTT |
| si-S-M#1 | UGCACCGCGAUGUAACUAATT |
| si-S-M#2 | CGGCGGUUGUUAGUUACAUTT |
| si-SLC2A11 | GGUGGGUACAGCCUGAUGATT |
| si-MIF | AUGAACAUCGGCAUGAUGGTT |
| si-PTBP1 | CCCUCAUUGACCUGCACAATT |
| si-CTCF#1 | GGUCUGCUAUCAGAGGUUATT |
| si-CTCF#2 | GCCUGCCGUAGAAAUUGAATT |
| si-NAT10 | CCAUCUCUCGCAUCUAUUUTT |
| **shRNA** |  |
| sh-NC | CAACAAGATGAAGAGCACCAA |
| sh-S-M#1 | TGCACCGCGATGTAACTAA |
| sh-S-M#2 | CGGCGGTTGTTAGTTACAT |

**Supplementary Table S2. Primers used in this study.**

| **Primer Name** | **Sequence 5’-3’** |
| --- | --- |
| **Used in qPCR** |  |
| *S-M* Forward | GGAAGCGAAGATCCAGTACG |
| *S-M* Reverse | GTGCACCGCGATGTAACTAA |
| *SLC2A11* Forward | TTATCATGGAGGCCTTGTCC |
| *SLC2A11* Reverse | GGATAACCTCCAGGCTCCTC |
| *MIF* Forward | CCGAGAAGTCAGGCACGTAG |
| *MIF* Reverse | GCACGTTGGTGTTTACGATG |
| *PTBP1* Forward | AGCGCGTGAAGATCCTGTTC |
| *PTBP1* Reverse | CAGGGGTGAGTTGCCGTAG |
| *PLK1* Forward | CACCAGCACGTCGTAGGATTC |
| *PLK1* Reverse | CCGTAGGTAGTATCGGGCCTC |
| *ROBO1* Forward | GACAAAACCCTTCGGATGTCA |
| *ROBO1* Reverse | CCAGTGGAGAGCCATCTTTCT |
| *PIK3R3* Forward | TACAATACGGTGTGGAGTATGGA |
| *PIK3R3* Reverse | TCATTGGCTTAGGTGGCTTTG |
| *CTCF* Forward | GGCATCGTCGTTACAAACACA |
| *CTCF* Reverse | TGGCATAACTGCACAAACTGC |
| *NAT10* Forward | AACGAGCTGGATTTGTTCCTG |
| *NAT10* Reverse | CTGGTCAGCCTCATCCTCATC |
| *GAPDH* Forward | GTCTCCTCTGACTTCAACAGCG |
| *GAPDH* Reverse | ACCACCCTGTTGCTGTAGCCAA |
| **Used in PCR** |  |
| *SLC2A11*-Exon8-Forward | GAAGCGAAGATCCAGTACGC |
| *SLC2A11*-Intron8-Reverse | TGGTTTAATGCAGAGGACCTG |
| **Used in RT** |  |
| *MIF-*Exon2 | TTGCTGTAGGAGCGGTTCTG |

**Supplementary Table S3. Antibodies used in this study.**

| **Application** | **Protein or histone modification** | **Manufacturer** | **Cat number** | **Dilution for usage** |
| --- | --- | --- | --- | --- |
| Western blotting | FLAG | CST | #86861 | 1:1000 |
| Western blotting | GAPDH | Proteintech | 60004-1-Ig | 1:10000 |
| Western blotting | PTBP1 | Abcam | ab133734 | 1:1000 |
| Western blotting | PLK1 | Proteintech | 10305-1-AP | 1:1000 |
| Western blotting | ROBO1 | Proteintech | 20219-1-AP | 1:1000 |
| Western blotting | PIK3R3 | Proteintech | 27035-1-AP | 1:5000 |
| Western blotting | Lamin B1 | Proteintech | 12987-1-AP | 1:1000 |
| Immunohistochemistry | Ki67 | ZSGB-BIO | ZM-0167-3 | 1:10 |
| Immunohistochemistry | Luciferase | Abcam | ab21176 | 1:1000 |
| Immunoprecipitation | IgG | CST | #3900 | 1:500 |
| Immunoprecipitation | FLAG | Proteintech | 66008-3-Ig | 1:50 |
| Immunoprecipitation | PTBP1 | Proteintech | 12582-1-AP | 1:50 |
| Immunofluorescence | FLAG | Proteintech | 66008-3-Ig | 1:100 |
| Immunofluorescence | PTBP1 | Proteintech | 12582-1-AP | 1:100 |
| Immunofluorescence | DAPI | Servicebio | G1012 | 1:10 |
| RIP | NAT10 | Abcam | ab194297 | 1:20 |
| RIP | N4-acetylcytidine | Abcam | ab252215 | 1:20 |
